# Ex vivo drug response profiling for response and outcome prediction in hematologic malignancies: the prospective non-interventional SMARTrial

**DOI:** 10.1038/s43018-023-00645-5

**Published:** 2023-10-02

**Authors:** Nora Liebers, Peter-Martin Bruch, Tobias Terzer, Miguel Hernandez-Hernandez, Nagarajan Paramasivam, Donnacha Fitzgerald, Heidi Altmann, Tobias Roider, Carolin Kolb, Mareike Knoll, Angela Lenze, Uwe Platzbecker, Christoph Röllig, Claudia Baldus, Hubert Serve, Martin Bornhäuser, Daniel Hübschmann, Carsten Müller-Tidow, Friedrich Stölzel, Wolfgang Huber, Axel Benner, Thorsten Zenz, Junyan Lu, Sascha Dietrich

**Affiliations:** 1 Department of Hematology, Oncology and Clinical Immunology, University Hospital Düsseldorf, Düsseldorf, Germany; 2 Department of Medicine V, Heidelberg University Hospital, Heidelberg, Germany; 3 Department of Translational Medical Oncology, National Center for Tumor Diseases (NCT) Heidelberg and German Cancer Research Center (DKFZ), Heidelberg, Germany; 4 Molecular Medicine Partnership Unit (MMPU), Heidelberg, Germany; 5 Center for Integrated Oncology Aachen-Bonn-Cologne-Düsseldorf (CIO ABCD), Aachen Bonn Cologne Düsseldorf, Germany; 6 Division of Biostatistics, German Cancer Research Center (DKFZ), Heidelberg, Germany; 7 Computational Oncology Group, Molecular Precision Oncology Program, NCT Heidelberg and DKFZ, Heidelberg, Germany; 8 European Molecular Biology Laboratory (EMBL), Heidelberg, Germany; 9 University Hospital TU Dresden, Dresden, Germany; 10 Leipzig University Hospital, Leipzig, Germany; 11 Department of Internal Medicine II, University Hospital of Kiel, Kiel, Germany; 12 Department of Internal Medicine II, University Hospital of Frankfurt Main, Frankfurt am Main, Germany; 13 Heidelberg Institute for Stem Cell Technology and Experimental Medicine, Heidelberg, Germany; 14 German Cancer Consortium (DKTK), Heidelberg, Germany; 15 Department of Medical Oncology and Hematology, Universitätsspital & Universität Zürich, Zürich, Switzerland; 16 The LOOP Zürich—Medical Research Center, Zürich, Switzerland; 17 Medical Faculty Heidelberg, Heidelberg University, Heidelberg, Germany

**Keywords:** Cancer, Haematological cancer, Cancer therapy

## Abstract

Ex vivo drug response profiling is a powerful tool to study genotype–drug response associations and is being explored as a tool set for precision medicine in cancer. Here we conducted a prospective non-interventional trial to investigate feasibility of ex vivo drug response profiling for treatment guidance in hematologic malignancies (SMARTrial, NCT03488641). The primary endpoint to provide drug response profiling reports within 7 d was met in 91% of all study participants (*N* = 80). Secondary endpoint analysis revealed that ex vivo resistance to chemotherapeutic drugs predicted chemotherapy treatment failure in vivo. We confirmed the predictive value of ex vivo response to chemotherapy in a validation cohort of 95 individuals with acute myeloid leukemia treated with daunorubicin and cytarabine. Ex vivo drug response profiles improved ELN-22 risk stratification in individuals with adverse risk. We conclude that ex vivo drug response profiling is clinically feasible and has the potential to predict chemotherapy response in individuals with hematologic malignancies beyond clinically established genetic markers.

## Abstract (web summary)

Liebers and colleagues present a prospective non-interventional trial in 80 individuals with hematologic cancer investigating the potential of ex vivo drug response profiling and show it to be clinically feasible.

## Main

Responses to anticancer treatments are often heterogeneous, and our understanding of factors that predict drug response is still unsatisfying. Advances have been made by using molecular phenotypes to predict drug response. In many hematologic malignancies, including acute myeloid leukemia (AML), acute lymphoblastic leukemia (ALL), chronic lymphocytic leukemia (CLL) and multiple myeloma, tailored mutation analysis and gene panel diagnostics have already become a routine procedure to subclassify risk groups and assign therapeutic strategies^1–5^. Furthermore, recent clinical studies suggested that precise genotype-informed treatment strategies can result in substantial survival benefits in individuals with AML or other rare types of cancer^6,7^. However, genomic profiling often does not reveal targetable mutations^8,9^. In addition, genome sequencing does not sufficiently explain the variance of drug response in all instances. For example, in CLL, other molecular omics layers, such as gene expression and DNA methylation, and their combination explained the variance of drug response to selected drugs noticeably better than genomic alterations alone^10^. Ex vivo drug response profiling (DRP) could integrate across this complex interplay of different molecular layers and could thereby serve as a complementary tool to genomic profiling for precision medicine.

Recently, the EXALT trial (ClinicalTrials.gov NCT03096821) demonstrated that ex vivo DRP can improve treatment guidance in individuals with advanced aggressive hematologic malignancies for whom standard therapies are not available^11^. In this study, ex vivo drug sensitivity was profiled in 143 human samples using a microscopy-based readout. Fifty-six individuals (39%) were treated according to the results of this assay, and 30 individuals (56%) benefited from this tailored treatment with a progression-free survival at least 30% longer than after the previous treatment. This proof-of-concept trial demonstrated the great potential of functional profiling for precision medicine. A second study measured ex vivo drug response profiles for 37 individuals with relapsed and refractory AML using an ATP-based assay with the aim to predict in vivo drug sensitivities. Again, this study demonstrated that individuals could benefit from such a functional precision medicine approach^12^. Both studies integrated their functional test results into a complex tumor board decision process, which supported the selection of a suitable personalized treatment for individuals with highly refractory disease courses who had failed standard treatments. These studies highlight the potential of functional precision medicine to guide treatment in individuals with highly refractory blood cancer. A systematic comparison of in vivo and ex vivo drug effects for standard treatment protocols across multiple hematologic disease entities was not performed in these studies.

Our prospective clinical non-interventional Systematic and Mechanism-Based Approach to Rational Treatment Trial of Blood Cancer (SMARTrial; ClinicalTrials.gov NCT03488641) complements recently published studies^11,12^ by demonstrating the feasibility of ex vivo DRP as a clinical routine test and by directly relating ex vivo and in vivo drug effects for standard treatment protocols and across multiple hematologic disease entities. In an independent and homogeneously treated validation cohort of 95 treatment-naive individuals with AML, we demonstrate that ex vivo DRP can improve genetic risk stratification.

## Results

### SMARTrial participants

**Fig. 1:**
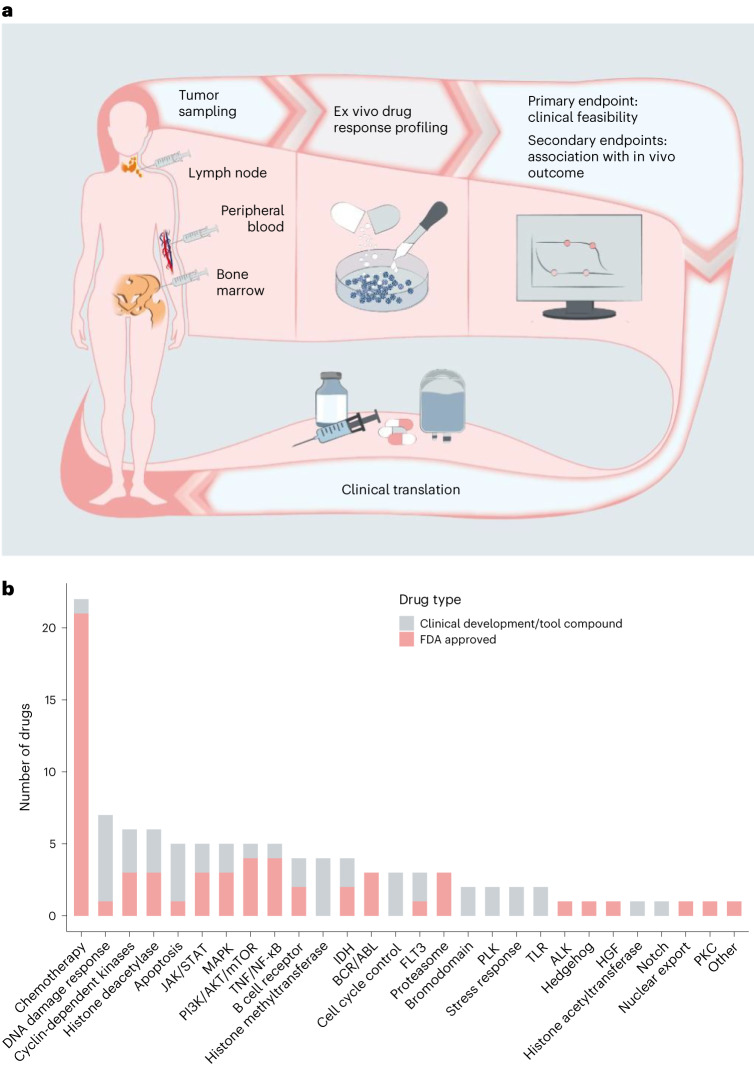
Overview of study design and drug selection. **a**, Outline of the prospective observational study. **b**, Overview of the drug classes in the compound library based on their targets/mode of action. FDA, Food and Drug Administration; TNF, tumor necrosis factor; IDH, isocitrate dehydrogenase; PLK, polo-like kinase 1; TLR, Toll-like receptor; HGF, hepatocyte growth factor; PKC, protein kinase C. Source data

**Fig. 2:**
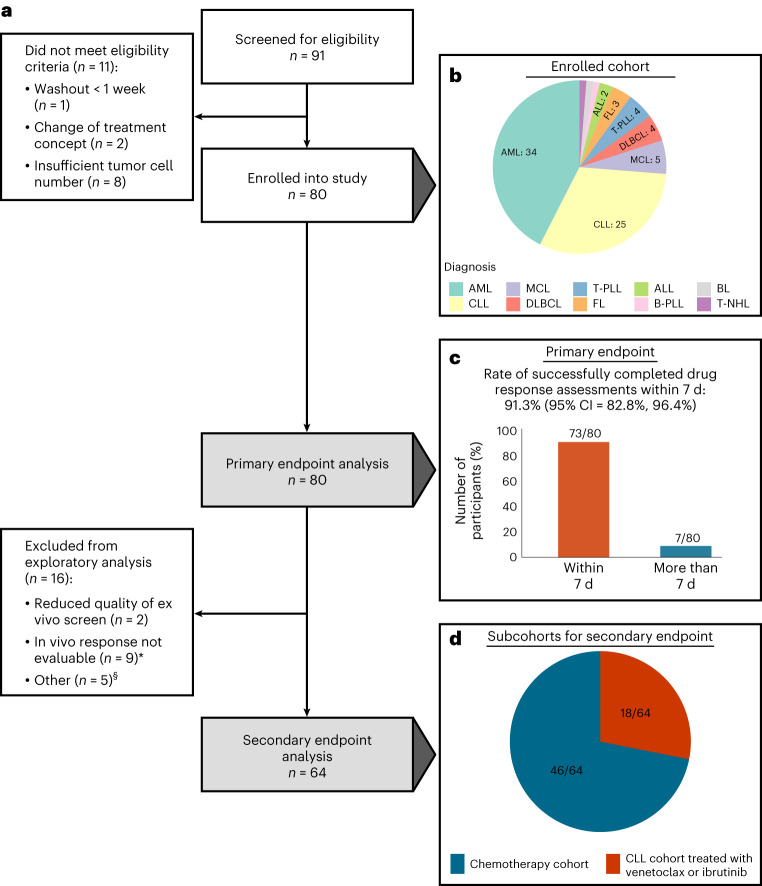
Flow diagram and primary endpoint analysis. **a**, Flow diagram of study participants and downstream analyses. The asterisk indicates that in nine individuals, the in vivo response was not evaluable due to the following reasons: scheduled treatment could not be initiated due to infection leading to death (*n* = 1), fulminant PD leading to early death (*n* = 1), scheduled treatment was denied by the participant before the start of treatment (*n* = 1) or within 14 d after treatment start (*n* = 1), scheduled treatment was prematurely discontinued due to side effects without prior response assessment (*n* = 2), scheduled treatment was discontinued several times due to side effects (*n* = 1) or response assessment was not available (*n* = 2). The section sign (§) indicates that five participants could not be assigned to the two subgroups (chemotherapy cohort and CLL cohort treated with venetoclax or ibrutinib) used for the secondary endpoint analysis. These participants were treated with palbociclib (*n* = 1), alemtuzumab (*n* = 1) and idelalisib ± rituximab (*n* = 2). One participant was treated with ibrutinib and rituximab but had a diffuse large B cell lymphoma. **b**, Enrolled participant cohort by diagnosis. **c**, Rate of successfully completed drug response assessments within 7 d (primary endpoint). **d**, Subcohorts for secondary endpoint analysis. MCL, mantle cell lymphoma; DLBCL, diffuse large B cell lymphoma; B-PLL, B cell prolymphocytic leukemia; T-NHL, T cell non-Hodgkin lymphoma. Source data

Between April 2018 and July 2020, 91 individuals with hematologic malignancies were screened for eligibility in the SMARTrial (Fig. 1). Eighty individuals (88%) fulfilled the eligibility criteria and were enrolled (Fig. 2a). The primary endpoint, ‘successful completion of the ex vivo drug response assay within 7 d’, could be assessed in all 80 individuals. For the secondary endpoint analysis, to systematically correlate ex vivo and in vivo drug responses, we assigned participants to two major subcohorts. Cohort 1 included individuals treated with chemotherapy (*n* = 46), and cohort 2 included individuals treated with venetoclax or ibrutinib (*n* = 18). Sixteen individuals were excluded from the secondary endpoint analysis because in vivo response to the prescribed treatment could not be assessed according to the study protocol (*n* = 9), ex vivo response profiles did not pass quality control (*n* = 2), or participants could not be assigned to either of the two subcohorts due to their prescribed therapies (*n* = 5).

Demographics and disease characteristics for all 80 eligible participants are shown in Table 1 and Supplementary Table 1. The median age was 68.5 years (range of 20–91 years). Participants with the following hematologic malignancies were included: AML (34/80, 42%), ALL (2/80, 2%), CLL (25/80, 31%), aggressive T cell leukemia (T cell prolymphocytic leukemia (T-PLL) 4/80, 5%), aggressive B cell lymphoma (5/80, 6%), aggressive T cell lymphoma (1/80, 1%) and indolent B cell lymphoma (9/80, 11%; Fig. 2b). The majority of participants were treatment naive (54/80, 68%), but individual participants were heavily pretreated with three or more prior lines of therapy before study entry (6/80, 8%). The median follow-up time of the study cohort was 2.1 years. Median time from diagnosis or determination of treatment indication to treatment initiation ranged from 2 d in T-PLL to 32 d in follicular lymphoma (FL; Supplementary Table 2).

**Table 1:** SMARTrial participant characteristics

Characteristics	Total (*N* = 80)
**Sex**	
Male	48 (60%)
Female	32 (40%)
**Age (years)**	
Median [minimum, maximum]	68.5 [20.0, 91.0]
**Diagnosis**	
AML	34 (42%)
ALL	2 (2%)
DLBCL	4 (5%)
BL	1 (1%)
FL	3 (4%)
MCL	5 (6%)
CLL	25 (31%)
B-PLL	1 (1%)
T-PLL	4 (5%)
T cell lymphoma, NOS	1 (1%)
**Time from initial diagnosis (months)**	
≤12	49 (61%)
>12	31 (39%)
**Prior lines of treatment**	
≥3	6 (8%)
0	54 (68%)
1	11 (14%)
2	9 (11%)
**Tumor sample origin**	
Peripheral blood	59 (74%)
Bone marrow	12 (15%)
Lymph node	9 (11%)
**Tumor infiltration of sample**	
Median [minimum, maximum]	84.5 [52.0, 99.0]
**Prescribed treatment at study entry**	
Chemotherapy	28 (35%)
Chemotherapy + small-molecule inhibitors	7 (9%)
Immunochemotherapy	16 (20%)
Immunotherapy	2 (2%)
Small-molecule inhibitors	27 (34%)

Data are number of participants (%) or median [range]. Percentages may not total 100 because of rounding.

NOS, not otherwise specified.

Human-derived primary tumor cells were obtained from peripheral blood (59/80, 74%), bone marrow aspirates (12/80, 15%) and lymph node biopsies (9/80, 11%). The median tumor purity of all 80 samples was 84.5% (range, 52–99%) and was assessed by immunophenotyping, peripheral blood smears or bone marrow cytology.

Components of almost all in vivo therapies (95%) recommended by the treating physician were included in our diagnostic ex vivo drug test. Sixty-four percent of participants (51/80) were scheduled for chemotherapy at study entry, either alone or in combination with immunotherapies and small-molecule inhibitors (Supplementary Table 3). Chemotherapy-free treatments, such as ibrutinib or venetoclax, were intended in 36% of participants (29/80). In total, three participants did not start the scheduled treatment due to fulminant progressive disease (PD), infection leading to death and refusal of the therapy by the participant after study inclusion.

### Feasibility of ex vivo DRP for clinical decision-making

The primary objective of the non-interventional SMARTrial was to evaluate the feasibility of a short-term ex vivo DRP assay for primary human-derived cancer cells in the clinical routine. Therefore, we evaluated the rate of successfully completed drug response assays within 7 d as the primary endpoint. The primary endpoint was met in 91.3% (95% confidence interval (95% CI) of 82.8–96.4%) of all eligible participants (Fig. 2c). The median time until the release of the final drug response report was 3 d (interquartile range (IQR) of 2–6 d, range of 2–17 d). The DRP was reported in the ‘SMARTrial explorer’, an interactive web application (http://mozi.embl.de/public/SMARTrial/), which could be accessed by the treating physicians. Reasons for delayed reporting in individual participants included suboptimal sample quality or study enrollment of participants before public holidays.

### Quality assessment

**Extended Data Fig. 1:**
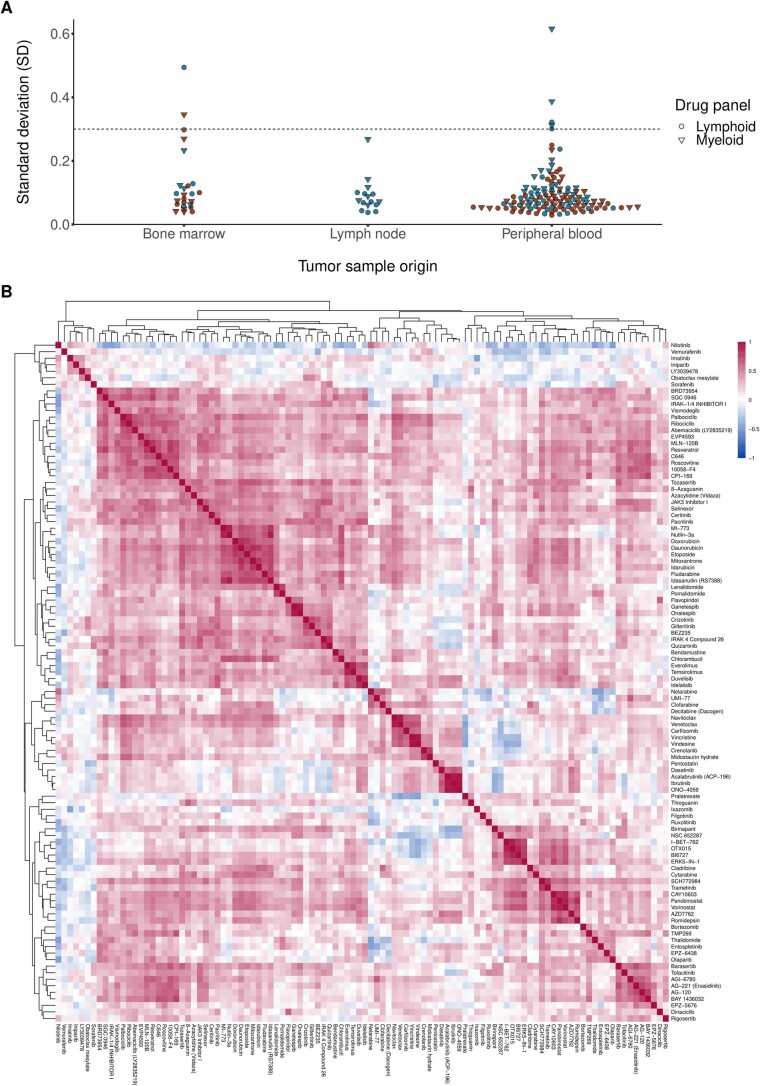
Quality assessment of SMARTrial cohort. (**a**) To estimate the technical variability of the performed assay, we calculated the standard deviation (SD) of all 16 inner DMSO controls for each individual drug plate per patient. Plates with a relatively high technical noise (SD of negative DMSO controls >0.3) were excluded from further analyses (excluded myeloid plates: S047, S050, S056, S062; excluded lymphoid plates: S047, S050, S062). For two patients (S047, S062), no additional tumor material was available for retesting and they were completely excluded from the subsequent analyses. (**b**) The heatmap shows the drug-drug correlations for all pairs of drugs. All patients with an evaluable drug response profiling were included (n = 78, exclusion of two patients with samples with relatively high technical noise). Pearson correlation coefficients were calculated from all ex vivo drug responses, measured as AUC of all concentrations per drug. Drug pairs with high correlation and anti-correlation are represented by red and blue squares, respectively. Drugs with similar mechanism strongly correlate with each other, for example BTK inhibitors: ibrutinib, tirabrutinib (ONO-4059), and acalabrutinib. Source data

We performed several steps of data quality assessment before the ex vivo DRP data were used for further exploratory analyses. First, we estimated the technical variability by calculating the standard deviation (s.d.) of 16 evenly distributed dimethyl sulfoxide (DMSO) controls for each drug plate per participant (Extended Data Fig. 1a). In general, the median s.d. was low (median s.d.: 0.08; range: 0.03–0.6), and we found no significant difference between the samples derived from different tumor sample origins (peripheral blood, bone marrow and lymph node; *P* = 0.86, one-way analysis of variance). Four myeloid and three lymphoma plates belonging to a total of four participants (S047, S050, S056 and S062) showed relatively high technical noise (s.d. of negative controls > 0.3). For two participants (S047 and S062), no additional tumor material was available for retesting, and these samples were therefore excluded.

**Extended Data Fig. 2:**
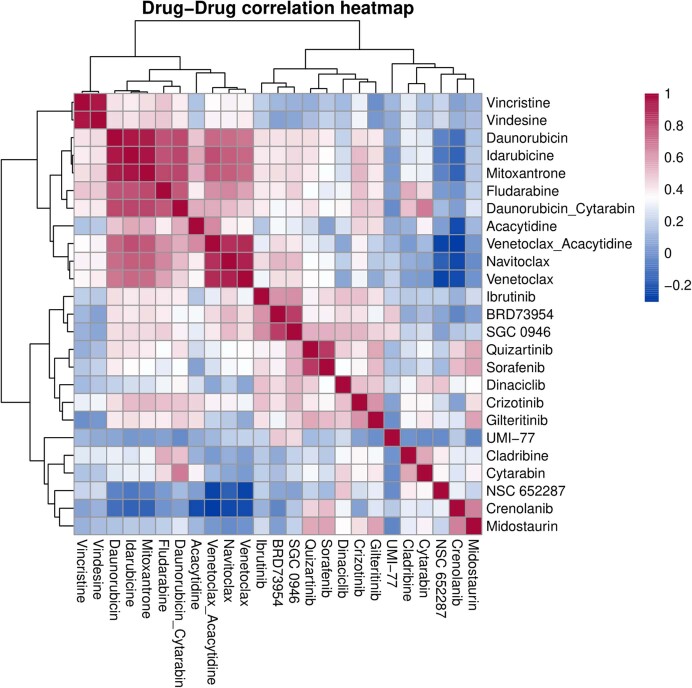
Drug-drug correlations of ex vivo drug responses – Validation cohort. The heatmap shows the drug-drug correlations for all pairs of drugs in all samples of the validation cohort (n = 95). Pearson correlation coefficients were calculated from all ex vivo drug responses, measured as AUC of all concentrations per drug. Drug pairs with high correlation and anti-correlation are represented by red and blue squares, respectively. Drugs with similar mechanism strongly correlate with each other, for example, navitoclax + venetoclax, vindesine + vincristine. The combination of daunorubicin and cytarabine correlates more strongly with daunorubicin than with cytarabine, indicating that the overall effect is more strongly driven by the toxicity caused by daunorubicin. Source data

**Extended Data Fig. 3:**
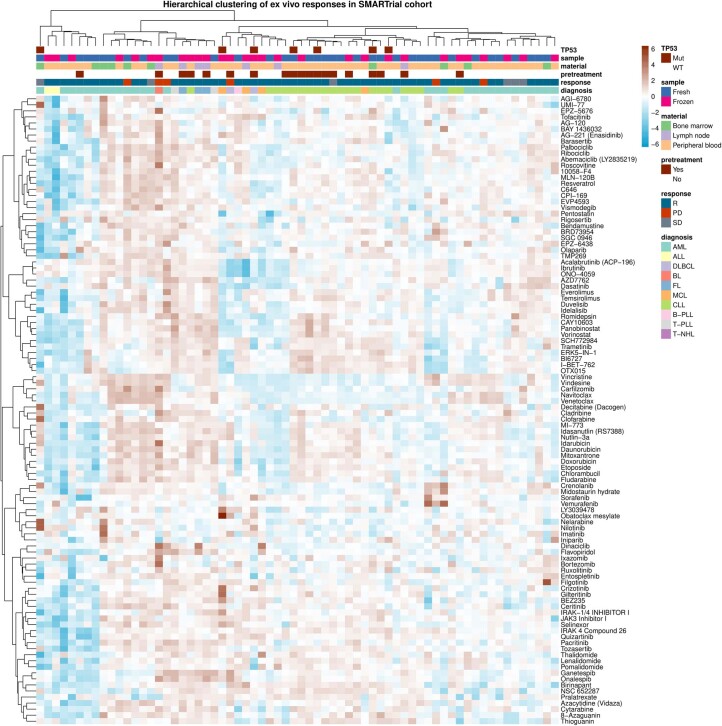
Drug response heatmap – SMARTrial. Heatmap showing ex vivo viability measurements after drug treatment for the SMARTrial cohort. The data are shown on a robust z-score scale, that is, the logarithm of the relative viability measurements was scaled by the median absolute deviation within each row. Red indicates increased viability, and blue indicates decreased viability. Samples are annotated for diagnosis, in vivo response, pre-treatment status, material type and TP53 mutation. n = 78. Source data

**Extended Data Fig. 4:**
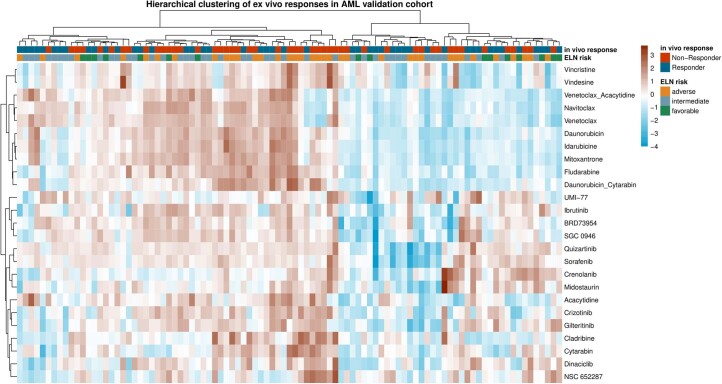
Drug response heatmap – validation cohort. Heatmap showing ex vivo viability measurements after drug treatment for the AML validation cohort. The data are shown on a robust z-score scale, that is, the logarithm of the relative viability measurements was scaled by the median absolute deviation within each row. Red indicates increased viability, and blue indicates decreased viability. Samples are annotated for ELN-22 risk groups and in vivo response. n = 95. Source data

**Extended Data Fig. 5:**
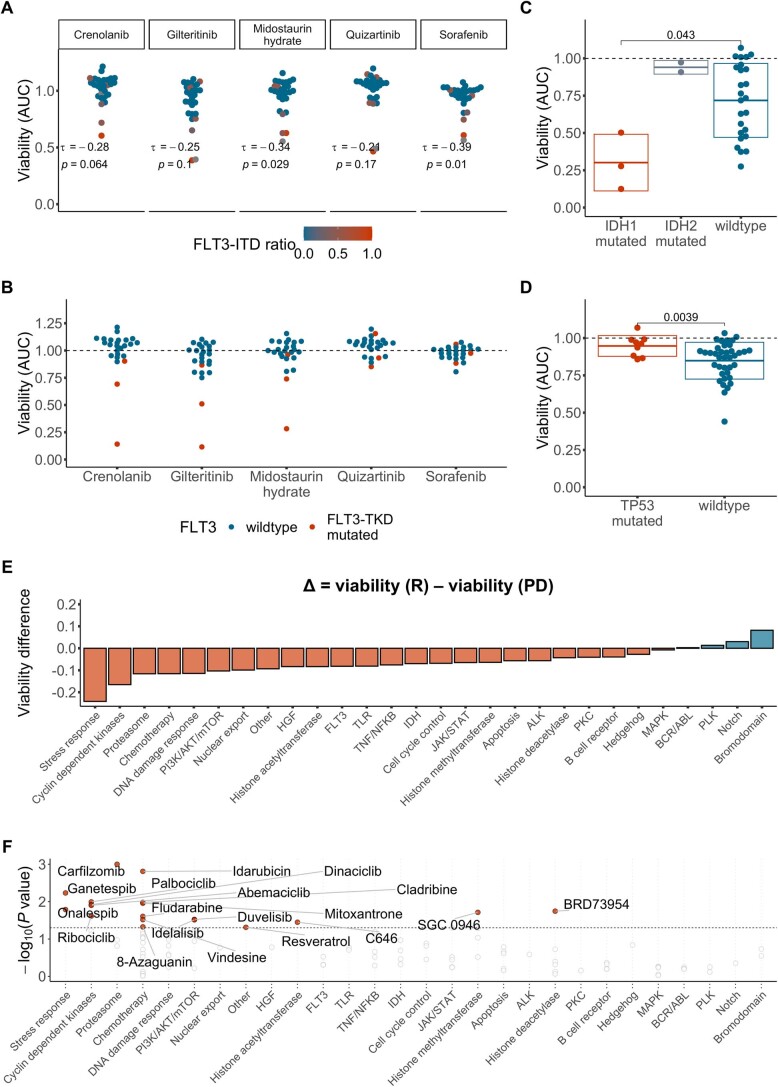
Association of ex vivo drug response phenotypes with genotype and clinical response groups. (**a**) Ex vivo drug responses to FLT3 inhibitors in AML samples (n = 27, excluding AML with FLT3 TKD mutation or missing FLT3 status). Kendall’s Tau for the correlation of ex vivo drug response and FLT3-ITD ratio shown. Ex vivo response calculated as averaged normalized viability across the 2 lowest concentrations. (**b**) Ex vivo response to FLT3 inhibitors in AML by FLT3-TKD mutation status (n = 24, excluding AML with missing FLT3 status or a FLT3-ITD mutation). Ex vivo response calculated as averaged normalized viability across the 2 lowest concentrations. (**c**) Ex vivo drug response to venetoclax in AML samples by IDH mutation status (n = 28, excluding AML with missing IDH status). Ex vivo response calculated as viability (AUC) across 5 concentrations. (**d**) Ex vivo drug response to nutlin-3a by TP53 mutation status (n = 42, SMARTrial samples from all entities, excluding samples with a missing TP53 status). (C + D) Each dot represents one patient sample, the boxes show mean +/− standard deviation. P value from two-sided Student’s t-test. Ex vivo response calculated as viability (AUC) across all 5 concentrations. (**e**) Ex vivo viability (AUC) per patient averaged by pathway and mean viabilities per pathway compared between chemosensitive patients (Clinical response (R): n = 34) and chemorefractory patients (Progressive disease (PD): n = 7) shown. Bars indicate mean viability difference between response groups. Red and blue indicates reduced and increased viability in chemosensitive samples, respectively. The bars are arranged by effect size and direction. (**f**) Significance in individual drug effect size between the clinical response groups. Student’s two sample t-tests were performed using ex vivo drug viability (AUC) between chemosensitive patients (Clinical response (R): n = 34) and chemorefractory patients (Progressive disease (PD): n = 7). The y axis shows the negative logarithm of the P values. Drugs are grouped by pathway and averaged effect size of the pathway group. P values smaller than the significance threshold (α = 0.05) are labeled. Source data

In addition, we assessed the validity of our ex vivo DRP pipeline by analyzing if expected drug–drug correlations and known gene–drug associations are recapitulated. Drugs with similar modes of action (for example, BTK inhibitors, BCL-2 inhibitors and vinca alkaloids) strongly correlated with each other (Extended Data Figs. 1b and 2), and participant samples clustered by diagnosis (Extended Data Figs. 3 and 4). Clinically well-established therapeutics recapitulated known vulnerabilities conferred by mutations, such as in *FLT3* and *IDH1*, in AML cells. For example, tumor cells with mutations in the *FLT3* tyrosine kinase domain (*FLT3*-TKD) were sensitive to the type I *FLT3* inhibitors crenolanib, gilteritinib and midostaurin, which bind the FLT3 receptor in the active conformation and are known to be active in AML cells with mutations in the *FLT3* internal tandem duplication (*FLT3*-ITD) and *FLT3*-TKD. By contrast, these tumor cells were insensitive to the type II FLT3 inhibitors quizartinib and sorafenib, which are known to be inactive in *FLT3*-TKD-mutated AML cells^13^ (Extended Data Fig. 5a,b). *IDH1*-mutated AML cells were specifically sensitive to venetoclax, which confirmed the known dependency of *IDH*-mutated AML on BCL-2 (ref. ^14^; Extended Data Fig. 5c). *TP53*-mutated tumor cells showed a decreased sensitivity to Nutlin-3a compared to wild-type tumor cells^10,15,16^ (Extended Data Fig. 5d). Drug responses can be explored in the interactive web application (http://mozi.embl.de/public/SMARTrial/).

### Association between ex vivo and in vivo responses

An important question of our study was to understand if ex vivo drug responses and in vivo responses correlate with each other. Considering the ongoing relevance of chemotherapy for hematologic tumors but also the increasing importance of chemotherapy-free targeted treatments in blood cancer, we focused on two subgroups: participants treated with chemotherapy (*n* = 46) and participants with CLL treated with venetoclax or ibrutinib (*n* = 18).

Among the 46 participants in the chemotherapy cohort, 29 were diagnosed with AML, 2 were diagnosed with ALL, and the remaining 15 were diagnosed with B or T cell lymphoma. All participants were treated with standard cytotoxic chemotherapy regimens combined with a targeted therapy (monoclonal antibodies or small-molecule inhibitors) in 46% of treatments.

**Fig. 3:**
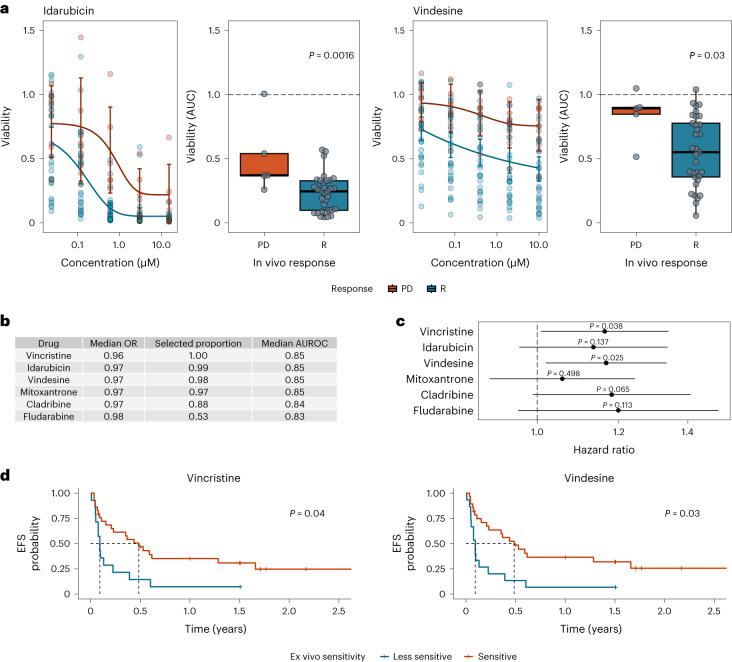
Association between ex vivo drug response and in vivo response or clinical outcome. **a**, Ex vivo sensitivity by clinical response group. Dose–response curves built by fitting a five-parameter logistic model using ex vivo viability measurements. Individual participant observations are displayed by circles in both plots. Blue and red represent groups of participants with clinical response (R) versus participants with PD, respectively (R: *n* = 33; PD: *n* = 5). Error bars represent mean and 95% CI. Centers, hinges and whiskers of the box plots signify medians, quartiles and 1.5× IQR, respectively. **b**, Elastic net logistic regression model of ex vivo drug viability (AUC) to chemotherapeutic agents with binary endpoint R versus PD (R: *n* = 33; PD: *n* = 5). The median odds ratio (OR) presented here relates to a change in ex vivo drug viability of 10%. Covariates are shown ordered by selection proportion (>0.5 shown here). The results of all covariates included in the model are shown in Supplementary Table 2. **c**, Association of ex vivo drug responses and EFS assessed by univariate Cox regressions (R: *n* = 33; SD: *n* = 5; PD: *n* = 5). Estimated hazard ratios with corresponding 95% CIs are shown. Ex vivo drug viability (AUC) was calculated per drug and scaled such that a unit change of the regressor corresponds to a 10% change in cell viability. *P* values are from two-sided Wald tests on Cox regression models. **d**, Kaplan–Meier plots for EFS stratified by ex vivo drug response to vincristine and vindesine (R: *n* = 33; SD: *n* = 5; PD: *n* = 5). Participant groups of ex vivo responders and weak responders were defined by ex vivo drug responses dichotomized using maximally selected log-rank statistics to visualize effects. Fourteen of 43 participants were classified as vincristine weak responders, and 15 of 43 participants were classified as vindesine weak responders. Source data

Our ex vivo drug screen covered a broad library of compounds with different modes of action. To determine which ex vivo drug response profiles were most suitable for predicting chemosensitivity in vivo, we chose an unbiased approach and associated all ex vivo drug response profiles with in vivo response categories (PD, stable disease (SD) and response (R)). Because direct comparisons between drugs were not feasible due to small numbers of uniformly treated participants and combination therapies in vivo, we grouped drugs according to their mode of action and associated the averaged ex vivo responses across all drugs within these classes with the in vivo response groups (R versus PD; Extended Data Fig. 5e). Ex vivo responses between chemosensitive and chemorefractory individuals differed most significantly for the following five drug classes: heat shock protein inhibitors (stress response), cyclin-dependent kinase inhibitors, proteasome inhibitors, chemotherapeutics and inhibitors involved in the DNA damage response signaling pathway. In a second step, we associated ex vivo drug response profiles of each individual drug with in vivo response (R versus PD; Extended Data Fig. 5f). Multiple drugs of the above-mentioned drug classes showed similar activity, suggesting that our data represent on-target effects as the primary mode of action of these drugs. Additional drugs with significantly different ex vivo drug sensitivities between responders and participants with PD were found among the groups of mTOR inhibitors, proteasome inhibitors and compounds that are involved in histone modifications. Representative examples of dose–response curves and averaged drug viabilities between individuals who were chemosensitive and chemorefractory are shown in Fig. 3a.

Individual ex vivo drug responses might not predict overall chemosensitivity. Therefore, we investigated how the combination of multiple ex vivo drug responses could be used to predict chemosensitivity. Because the majority of relevant drugs with significant differences were found among the chemotherapeutics (the treatment most individuals received in vivo), we focused on the group of chemotherapeutics to avoid overfitting. For variable selection, we built an elastic net logistic regression model and regressed ex vivo drug response profiles of individual chemotherapeutic agents to in vivo response (R versus PD; Fig. 3b and Supplementary Table 4). In total, we fitted 1,000 models based on different randomly selected folds. In more than 80% of all models, both vinca alkaloids, vincristine and vindesine, the two anthracyclines idarubicin and mitoxantrone and the purine analog cladribine were selected as prognostic features. Our models including the five above-mentioned chemotherapeutics reached a median cross-validation area under the receiver operating characteristic curve (AUROC) of 0.84 to 0.85, highlighting the discriminative ability of these models.

To elucidate the predictive power of features selected by the elastic net regression model on the durability of achieved clinical responses, we regressed event-free survival (EFS) on these drug response profiles. An event was defined as PD, change of treatment or death. We found that stronger ex vivo responses to both vinca alkaloids were associated with extended EFS in the chemotherapy cohort (Fig. 3c,d). Together, these data suggest that ex vivo drug response phenotyping is useful in predicting important clinical endpoints in individuals treated with chemotherapy across hematologic malignancies.

**Extended Data Fig. 6:**
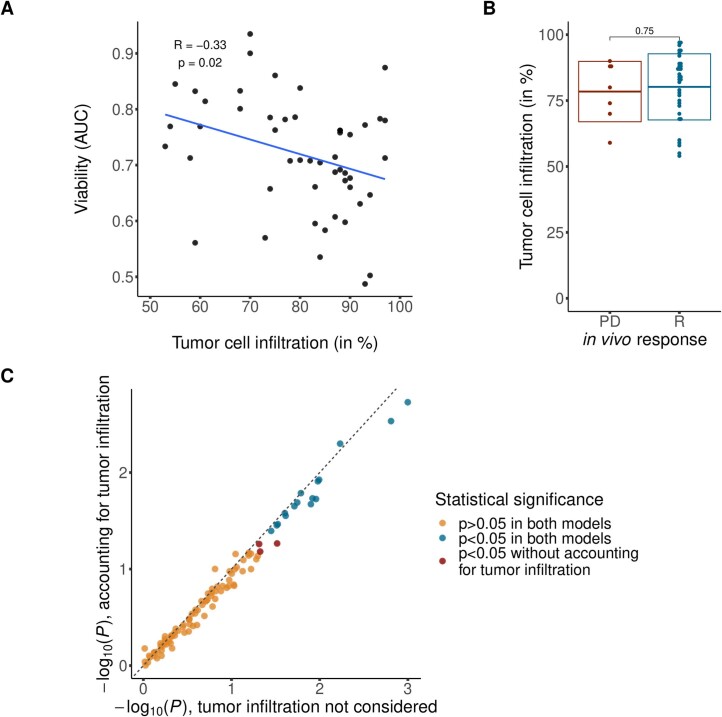
Tumor cell infiltration. (**a**) Association between tumor cell infiltration and ex vivo drug response to chemotherapeutic agents in patients treated with chemotherapy. Ex vivo drug response per patient was averaged within the group of chemotherapeutic agents. Pearson correlation coefficient and respective P value from two-sided Pearson correlation shown in the plot. (**b**) Tumor cell infiltration stratified by in vivo response groups. Each dot represents one patient sample, the boxes show mean +/− standard deviation. P value from two-sided Student’s t-test. (Clinical response (R): n = 34, Progressive disease (PD): n = 7). (**c**) Significance in individual drug effect size between the clinical response groups as shown in Fig. 3b. P values from two-sided Student’s t-test with (y-axis) and without (x-axis) taking tumor infiltration into account. Source data

We further investigated if the tumor cell infiltration across the chemotherapy cohort, which ranged from 54 to 97%, had an impact on the observed ex vivo response to chemotherapeutic agents and may have confounded the ex vivo–in vivo drug response association. We observed a weak correlation between tumor cell infiltration and ex vivo response to chemotherapeutic agents (*r* = −0.33, *P* = 0.02; Extended Data Fig. 6a) but no correlation between tumor cell infiltration and in vivo response (Extended Data Fig. 6b). Furthermore, we used tumor infiltration as a blocking factor that did not affect the significance of most ex vivo–in vivo drug response associations (Extended Data Fig. 6c). We conclude that ex vivo–in vivo drug response associations for chemotherapeutic agents are not confounded by tumor infiltration rate in our study.

**Extended Data Fig. 7:**
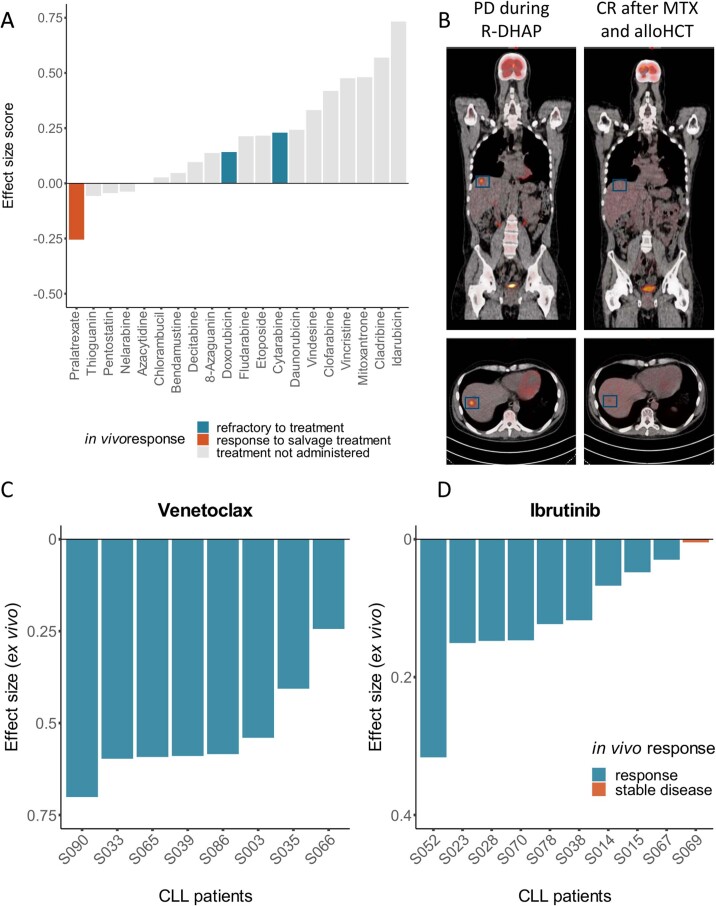
Clinical translation of ex vivo drug response profiling in a patient with highly aggressive refractory Burkitt lymphoma. (**a**) Normalized ex vivo effect size scores (ESS) of all chemotherapeutics for S005. The effect size (1- viability AUC) of each drug was subtracted from the median effect sizes of the same drug of the complete SMARTrial study cohort. A negative and positive ESS indicate a higher or lower effect size in this specific patient sample, respectively. S005 was less responsive to the majority of chemotherapeutics than the cohort median ex vivo. The ex vivo effect of pralatrexate was higher than the median effect across all SMARTrial samples. (**b**) PET-CT scans of participant S005. Left: PET-CT scan showing a progressive disease (PD) in the liver after previous treatment with R-DHAP (rituximab, high dose cytarabine, cisplatin, dexamethasone). Right: PET-CT scan after treatment with 3 cycles of methotrexate (MTX) and consolidating allogeneic hematopoietic cell transplantation (alloHCT). The patient achieved a partial response after treatment with MTX which enabled him to undergo alloHCT and eventually resulted in complete remission. (C + D) Association of in vivo and ex vivo response in CLL patients treated with venetoclax +/- anti- CD20 treatment (**c**) and ibrutinib (**d**). The ex vivo drug effect size is defined as 1-viability(AUC). Each bar represents one patient. The color of the bar represents in vivo response. Source data

Although this study was a non-interventional study and individuals were treated according to the treatment that was scheduled by the treating physician before study entry, one participant (S005) received ex vivo drug response-informed treatment after failure of all standard chemotherapies. This participant suffered from refractory Burkitt lymphoma (BL). Lymphoma cells were insensitive to almost all drugs in the drug screen (Extended Data Fig. 7a). However, in the drug ranking, we identified a strong ex vivo sensitivity to pralatrexate. This suggested that the participant might benefit from treatment with a folate antagonist. The participant agreed to this individual ex vivo-guided treatment approach, and after three cycles of high-dose methotrexate, the participant achieved a partial response. Subsequently, the participant underwent a consolidating allogeneic stem cell transplantation, which resulted in complete remission (Extended Data Fig. 7b). This example illustrates how ex vivo DRP can reveal unexpected effective anticancer drugs and support treatment decisions, especially in individuals for whom standard treatments are no longer available.

We further investigated the association between ex vivo and in vivo responses of targeted therapies using the example of individuals with CLL. Our study cohort included eight individuals who were treated with venetoclax and ten individuals who were treated with ibrutinib. Venetoclax was combined with an anti-CD20 treatment in the majority of participants (seven of eight). Concordant with the in vivo response, the primary tumor cells of all participants who were treated with venetoclax showed ex vivo sensitivity to venetoclax (Extended Data Fig. 7c). Ibrutinib exhibited smaller effect sizes than venetoclax in our short-term ex vivo assay, which is in line with clinical response dynamics and previous studies^10^. Cells from the only participant who showed insufficient in vivo efficacy of ibrutinib exhibited the weakest ex vivo response to ibrutinib (S069; Extended Data Fig. 7d). This participant had received ibrutinib before, which was discontinued due to atrial fibrillation. A genetic profiling of this participant’s tumor cells was performed, but no mutation known to confer ibrutinib resistance was found, demonstrating the potential to improve response prediction beyond known genetic risk markers.

### Validation of ex vivo and in vivo response association

**Fig. 4:**
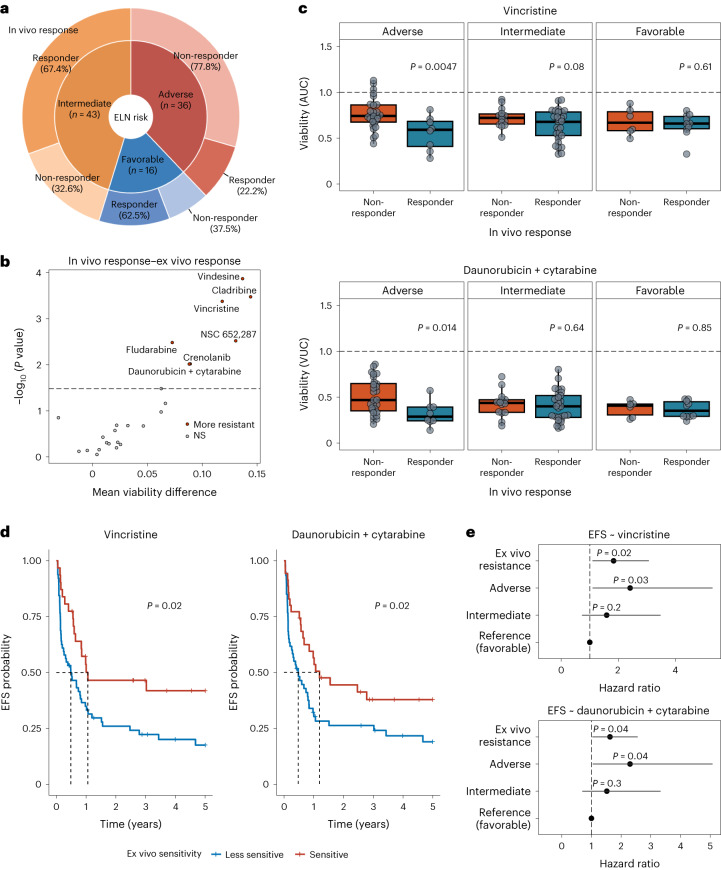
Validation of the association between ex vivo and in vivo drug responses in a cohort of individuals with AML. **a**, Overview of the validation cohort. The inner circle represents ELN-22 risk groups in total numbers, and the outer circle represents the distribution of in vivo responders and non-responders in ELN-22 risk groups. **b**, Ex vivo treatments with significantly different responses in in vivo responders (*n* = 47) and non-responders (*n* = 48). Negative log_10_ (*P* value) of Student’s *t*-tests is shown on the *y* axis, and mean difference between responders and non-responders is on the *x* axis. The dashed line represents the 10% false discovery rate cutoff (Benjamini–Hochberg procedure); NS, not significant. **c**, Viability (AUC) after ex vivo treatment with vincristine (top) and viability (volume under the curve (VUC)) after treatment with daunorubicin and cytarabine (bottom) separated by ELN-22 risk groups (ELN-22 adverse risk: non-responder: *n* = 28, responder: *n* = 8; ELN-22 intermediate risk: non-responder: *n* = 14, responder: *n* = 29; ELN-22 favorable risk: non-responder: *n* = 6, responder: *n* = 10). *P* values are derived from two-sided Student’s *t*-tests. Centers, hinges and whiskers of the box plots signify medians, quartiles and 1.5× IQR, respectively. **d**, Kaplan–Meier plots for EFS stratified by ex vivo drug response to vincristine and daunorubicin + cytarabine. For visualization purposes, participant groups of ex vivo responders and weak responders were defined by ex vivo drug responses dichotomized using maximally selected log-rank statistics to visualize effects. Sixty-four of 95 participants were classified as vincristine weak responders. Sixty of 95 participants were classified as daunorubicin + cytarabine weak responders. *P* values are from two-sided Wald tests on Cox regression models using drug responses as continuous variables. **e**, Forest plot of hazard ratios in multivariate Cox proportional hazards models for EFS including ELN-22 risk groups and viability after ex vivo treatment with vincristine (top) and daunorubicin + cytarabine (bottom). The ex vivo responses (AUC values) were centered by mean and scaled by 2 s.d. to bring them to a similar scale as the categorical ELN-22 risk group variables. Source data

We further aimed to investigate if ex vivo DRP may improve clinical standard genetic risk profiling. Therefore, we focused on first-line treatment of AML, where genetic risk profiling is considered clinical standard. We assembled a validation cohort of 95 clinically well-annotated AML biosamples from the AML biobank of the German Study Alliance for Acute Myeloid Leukemia (SAL). All biosamples were obtained from treatment-naive participants with AML who were scheduled to receive induction therapy with daunorubicin and cytarabine. The cohort was compiled to contain 47 responders and 48 non-responders to induction therapy from all ELN-22 risk groups (Fig. 4a, Table 2 and Supplementary Table 5).

**Table 2:** Validation cohort participant characteristics

Characteristics	Total (*N* = 95)
**Sex**	
Female	47 (49%)
Male	48 (51%)
**Age (years)**	
Median [minimum, maximum]	59.0 [18.0, 84.0]
**Diagnosis**	
AML	95 (100%)
**Tumor cell infiltration**	
Median [minimum, maximum]	76.0 [50.0, 99.0]
**Prescribed treatment**	
Cytarabine + daunorubicin	95 (100%)
**In vivo** **response group**	
Non-responder	48 (51%)
Responder	47 (49%)
**ELN-22 risk group**	
Adverse	36 (38%)
Favorable	16 (17%)
Intermediate	43 (45%)

Data are number of participants (%) or median [range]. Percentages may not total 100 because of rounding.

**Extended Data Fig. 8:**
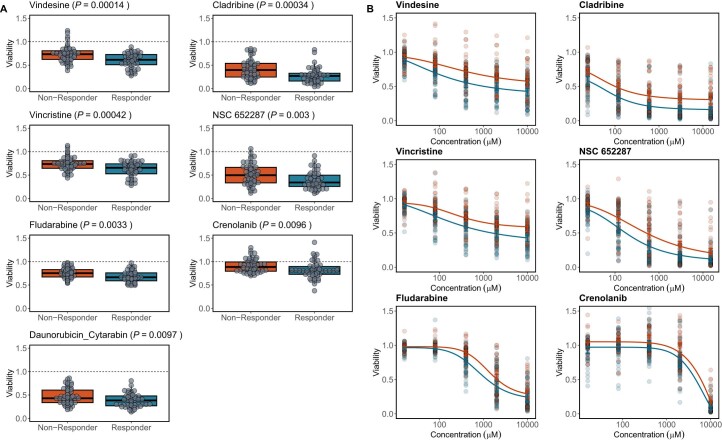
Significantly different ex vivo drug responses between responders and non-responders. (**a**) Boxplots showing viability (AUC in single-drug conditions and VUC in daunorubicin + cytarabine combination) after ex vivo drug treatment for drugs with significantly different responses in responders and non-responders. P-values from two-sided Students t-tests. Responder: n = 47, Non-responder: n = 48. Center, hinges and whiskers of the boxplots signify median, quartiles and 1.5x IQR, respectively. (**b**) Dose response curves for corresponding single drug conditions. Dose-response curves were built by fitting a 5-parameter logistic model for each clinical response group using ex vivo viability measurements across five drug concentrations. Individual patient observations are displayed by circles. Error bars represent mean and 95% CI. Responder: n = 47, Non-responder: n = 48. Source data

As a first step, we compared ex vivo drug response in the validation cohort between clinical responders and non-responders independently of genetic risk profiles. Ex vivo drug response profiles differed significantly between in vivo responders and non-responders (Benjamini–Hochberg-adjusted^17^ *P* value of <0.1). Interestingly, clinical in vivo response to induction therapy with daunorubicin and cytarabine was significantly associated with ex vivo response to the combination treatment with daunorubicin and cytarabine but not with ex vivo response to either drug alone. Vincristine, vindesine and cladribine, which were among the strongest predictors of in vivo response in the elastic net logistic regression model in the SMARTrial cohort, again showed the strongest association between in vivo and ex vivo response in the validation cohort (Fig. 4b and Extended Data Fig. 8).

**Extended Data Fig. 9:**
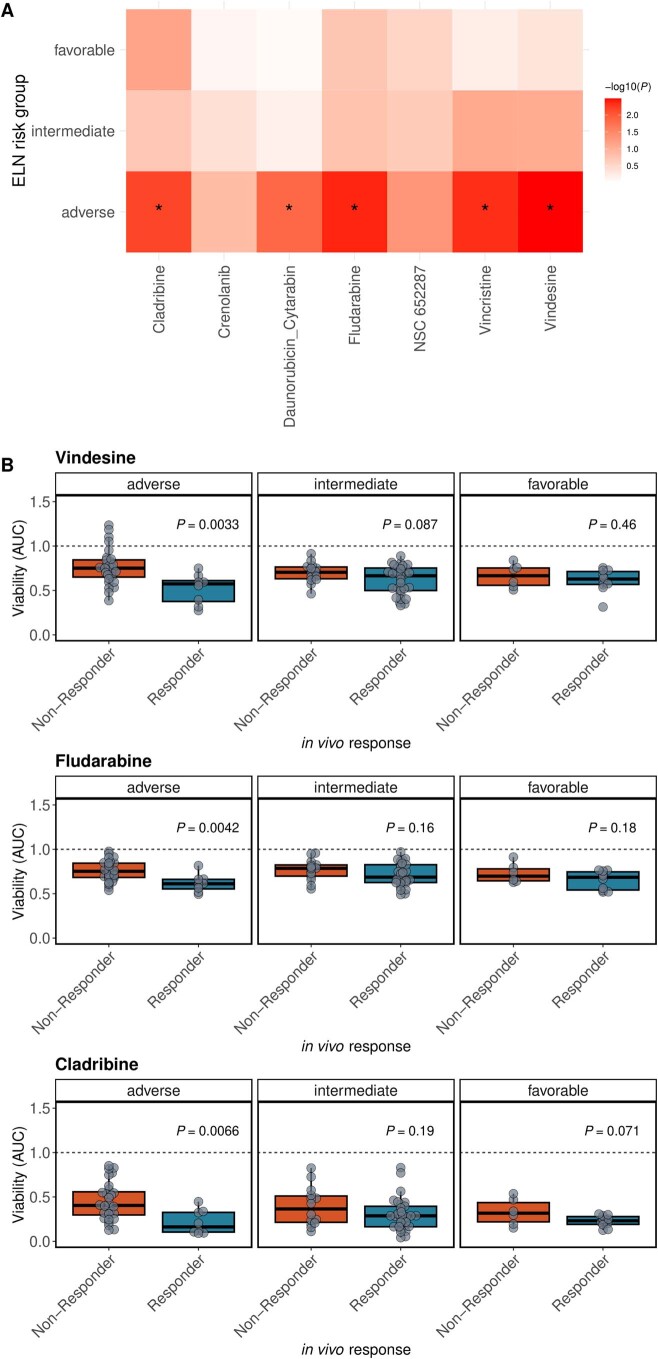
Significantly different ex vivo drug responses between responders and non-responders in ELN-22 risk groups. (**a**) Heatmap of p-values from two-sided Student’s t-tests between ex vivo drug response and in vivo therapy response. BH-adjusted p-values below 0.1 are marked with an asterisk. (**b**) Boxplots showing viability (AUC) after ex vivo drug treatment for drugs with significantly different responses in responders and non-responders in ELN-22 risk subgroups. Corresponding to Fig. 4c. P-values from two-sided Students t-tests. Responder: n = 47, Non-responder: n = 48. Center, hinges and whiskers of the boxplots signify median, quartiles and 1.5x IQR, respectively. Source data

As a next step, we investigated whether ex vivo DRP could further improve clinical in vivo response prediction and compared drug response profiles within each ELN-22 risk group, a well-established and very recently updated AML risk stratification tool^4^. Ex vivo response profiles significantly distinguished in vivo responders to daunorubicin and cytarabine from non-responders in the genetic adverse risk group defined as per ELN-22 recommendations^4^ (Fig. 4c and Extended Data Fig. 9).

**Extended Data Fig. 10:**
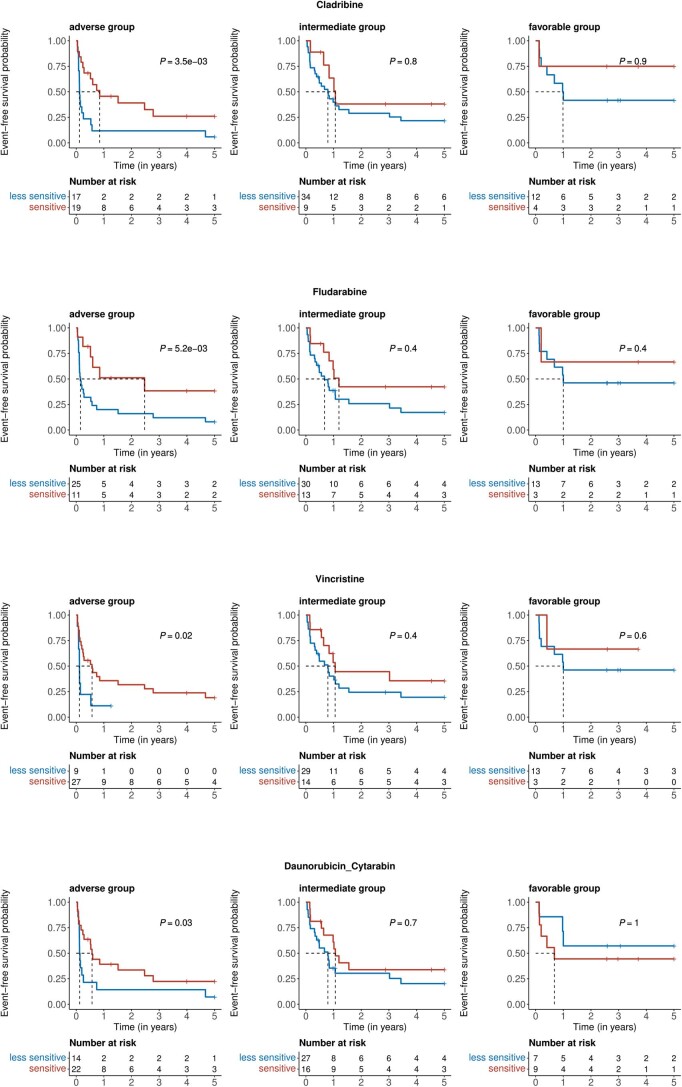
Significant associations of ex vivo drug responses and event free survival in ELN-22 risk groups. Kaplan-Meier plots for event-free survival stratified by ex vivo drug response to cladribine, fludarabine, vincristine and daunorubicin + cytarabine and faceted by ELN-22 risk groups. Patient groups of ex vivo responders and weak responders were defined by ex vivo drug responses dichotomized using maximally selected log rank statistics to visualize effects. Absolute numbers per groups are shown in tables below the plots. P-values are from two-sided Wald tests on Cox regression models using drug responses as continuous variables. Source data

In addition, we regressed ex vivo drug response profiles on EFS to assess the ability of ex vivo drug response to predict durability of achieved responses. Indeed, poor ex vivo drug response to vincristine as well as daunorubicin and cytarabine was associated with adverse EFS in participants with AML after induction therapy with daunorubicin and cytarabine (Fig. 4d). Ex vivo DRP further improved outcome prediction especially in participants with adverse risk as determined by ELN-22 (Fig. 4e and Extended Data Fig. 10). These results suggest that ex vivo DRP may improve in vivo response prediction beyond established genetic risk stratification tools in AML.

## Discussion

Although precision cancer medicine was almost synonymous with genome-informed cancer treatment for many years, functional profiling is now gaining importance and is transforming our understanding of precision cancer medicine^8^.

Genome-informed precision medicine relies on previously identified and well-characterized genotype–drug response associations and the identification of actionable mutations. Therefore, treatment recommendations can only be derived in a fraction of individuals with cancer^7–9^. By contrast, functional profiling can provide information about presumably effective treatments even in the absence of known targetable genetic lesions^11,12^. In addition, functional profiling can also reveal drug resistance in the absence of known genetic resistance markers.

Two recently published key studies have shown that functional profiling can successfully guide treatment recommendations in individuals with hematologic malignancies refractory to standard treatments^11,12^. These studies incorporated functional profiling in a tumor board decision process and produced treatment recommendations beyond established treatment options. In the aforementioned EXALT trial, 54% of the participants treated according to the functional precision medicine results achieved a progression-free survival at least 30% longer than the duration of that from prior therapy^11^. Although a direct comparison to a control arm was not planned, this response was improved compared to the participants treated according to physicians’ choices. These results highlight the potential of functional profiling to guide treatment in individuals with highly refractory disease.

Our prospective non-interventional SMARTrial was designed to explore the feasibility of ex vivo DRP and its value as a potential predictive tool in standard treatment settings. We demonstrated that functional profiling by high-throughput ex vivo drug testing is clinically feasible in individuals with blood cancers. The median turnaround time of 3 d makes it particularly useful for aggressive and rapidly progressing hematologic malignancies such as AML or aggressive lymphomas. A combination of five chemotherapeutic agents with different modes of action was able to read out the in vivo response to chemotherapy. In particular, ex vivo responses to vincristine and vindesine were significantly associated with EFS in individuals treated with chemotherapy.

The direct association of ex vivo and in vivo drug response was limited by the number of participants who received homogeneous in vivo treatments in the SMARTrial. We validated the predictive value of ex vivo chemotherapy drug response profiles in an independent cohort of individuals with AML who homogeneously received first-line treatment with daunorubicin and cytarabine. In both cohorts, we observed that ex vivo response to vindesine, vincristine and cladribine separated in vivo responders from non-responders, suggesting that these drugs are very suitable to read out in vivo chemotherapy resistance. Furthermore, in the homogeneously treated AML cohort, the in vivo response to daunorubicin and cytarabine correlated with ex vivo response to the same drugs, highlighting the direct association between in vivo and ex vivo response.

The strongest association between in vivo and ex vivo response was observed in individuals with AML with adverse risk according to the ELN-22 classification. A possible explanation for this observation might be that the ex vivo DRP assay reads out tumor cell-intrinsic mechanisms of resistance and sensitivity mediated by non-genetic risk factors or rare genetic events not considered in the ELN-22 risk classification. These results suggest that functional tests could serve as a surrogate for multiple resistance mechanisms mediated by different omics layers.

However, there was no significant difference in ex vivo response profiles between clinical responders and non-responders in the favorable ELN-22 risk group, although 6 of 16 participants in the low-risk group did not respond to treatment in vivo. This could be a result of insufficient statistical power and needs to be investigated in future studies.

Viable tumor cells are required to perform an ex vivo drug response assay. In our study, the availability of 5 × 10^7^ viable tumor cells was an inclusion criterion. In the EXALT trial, 20 of 143 tested participants were excluded due to insufficient tumor material^11^. This limits the applicability in the clinical context because not all individuals can be profiled ex vivo. Further, a potential bias can be introduced if the inability to obtain sufficient tumor material is linked to biological phenotypes, such as more aggressive disease courses (that is, higher content of leukemic cells in peripheral blood) or the infiltration of tumor tissue by stromal cells. Lastly, hematologic diseases, such as hairy cell leukemia or multiple myeloma, with typically low amounts of obtainable tumor cells are difficult to investigate using functional studies. Miniaturized profiling approaches, which require much smaller amounts of tumor cells^18,19^, have the potential to alleviate this limitation.

Additional limitations of our short-term ex vivo DRP assay are the inability to profile small subpopulations of therapy-resistant AML cells, which could give rise to relapse in vivo after being clonally selected, and the inability to consider microenvironmental factors, which may have an influence on in vivo drug response. Test assay modifications that address these limitations might be necessary to improve prediction accuracy.

In conclusion, our study demonstrates that ex vivo DRP is clinically feasible and can be used to predict in vivo response to standard treatment beyond known genetic risk factors. Further studies that reduce technical hurdles and establish reliable prognostic models for anticancer treatments are warranted and have the potential to improve therapy response and reduce side effects by ineffective treatments.

## Methods

### Study design

The SMARTrial was a single-center, prospective non-interventional study of ex vivo drug screening in hematologic malignancies. The study complied with all relevant ethical regulations and was approved by the ethics committee of the University of Heidelberg (S-683/2016) and conducted in accordance with the Declaration of Helsinki. All participants provided written informed consent. Participants were enrolled between April 2018 and July 2020. The last end-of-study visit was in July 2021.

Adult participants with a diagnosis of a hematologic malignancy in need of treatment and willing to donate sufficient tumor material were eligible. Additional eligibility criteria included measurable disease burden for response assessment and the availability of at least 5 × 10^7^ cells from peripheral blood draws, bone marrow aspirates or lymph node biopsies. Systemic cancer treatment other than cytoreductive pretreatment within 7 d before enrollment was an exclusion criterion. Complete inclusion and exclusion criteria are available in the study protocol (Supplementary Information). The primary endpoint was defined as the rate of successfully completed assessments of drug response within 7 d. The secondary endpoints included the accuracy of participant drug response prediction by ex vivo DRP and the prediction of clinical outcome parameters. Demographic and clinical data were collected at study entry. During the treatment and post-treatment phases, participants were regularly assessed (at weeks 1–4 and at months 3, 6 and 12 after treatment cessation or change) for at least 1 year after study entry. Clinical data were recorded in an electronic case report form using Onkostar (IT-Choice Software).

Because this study was a non-interventional study, participants were treated according to the treatment that was scheduled by the treating physician before study entry. However, one participant (S005) failed to respond to all standard chemotherapies (including the scheduled treatment before study entry). The ex vivo drug screening of this participant’s tumor cells revealed a sensitivity of the tumor cells to a chemotherapy that is approved for the participant’s type of cancer. The according treatment was recommended as an individual treatment protocol, and the participant provided informed consent before treatment.

For the validation cohort, we investigated the drug responses of 95 AML samples obtained from the AML biobank of the SAL (EK 98032010). All participants consented to biobanking and sample use for research projects.

### Ex vivo DRP

#### Samples

Tumor samples from different origins, including peripheral blood, bone marrow and lymph nodes, were included in the SMARTrial. Mononuclear cells were isolated from peripheral blood and bone marrow samples using a Ficoll gradient (GE Healthcare) and either used fresh or cryopreserved until further processing. Lymph node samples from participants were processed as previously described^20^. The samples within the validation cohort were taken from participants with AML at initial diagnosis, isolated from bone marrow aspirates using a Ficoll gradient (GE Healthcare) and cryopreserved.

#### Compounds and drug plates

Ex vivo responses to 106 drugs were measured (Supplementary Table 6) in the SMARTrial. Compounds were obtained from Selleck Chemicals, Sigma-Aldrich, Merck, BioCat and Biomol, were dissolved in DMSO (Serva) at 1–205 mM (mainly 10–25 mM) and stored at –20 °C. We preplated compounds in 384-well plates (Greiner Bio-One, 781904), which were sealed with silver foil (Greiner Bio-One, 676090). These plates were stored as ‘ready-to-use plates’ at –20 °C until needed. Due to the large number of compounds, we had two sets of plates containing drug panels for lymphoid or myeloid diseases. If the final cell count of a sample was only sufficient for one drug panel, we selected the appropriate panel based on the disease entity. On each plate, we used one well per drug and concentration. Each plate contained 63 compounds with five concentrations per compound based on previous experiments^10^. Twenty compounds were considered important for both lymphoid and myeloid samples and were plated on both plates. Sixty-four DMSO solvent controls were evenly distributed over the full plate.

#### Drug response assay

For the drug response assays, we used RPMI-1640 (Gibco, 21875-034) supplemented with 1% penicillin/streptomycin (Gibco, 15140-122), 1% l-glutamine (Gibco, 25030-024) and heat-inactivated human serum (Sigma, H6914-100ml). For frozen samples, samples were thawed as previously reported^21^, DMSO was removed, and the cells were incubated in cell culture medium at room temperature for 3 h on a roll mixer. Fresh samples were directly processed after isolation of mononuclear cells. The final cell number was 4 × 10^4^ cells per well. The ATP-based CellTiter Glo assay (Promega) was used to determine cell viability after an incubation period of 48 h at 37 °C and 5% CO_2_. After 20 min of incubation with the CellTiter Glo reagent, luminescence was measured with an EnSight Multimode plate reader (PerkinElmer). The integration time was 0.1 s per well. Due to ex vivo inefficacy of cyclophosphamide and incompatibility of cisplatin with DMSO, both drugs were excluded from further downstream analyses. The drug response assay for the validation cohort was performed as detailed above with the following changes. In total, 24 drugs were screened at five concentrations each (Supplementary Table 7). Daunorubicin and cytarabine as well as venetoclax and azacytidine were screened both as single drugs and in combination in the validation cohort. Each participant sample in the validation cohort was screened in 192 wells of a 384-well plate (Greiner Bio-One, 781904). For each sample in the validation cohort, 26 DMSO solvent controls were measured.

### In vivo response assessment

#### SMARTrial

At study entry, a main disease-specific response parameter was defined for each participant for response evaluation during study follow-up. These disease-specific response parameters were (1) blood counts, (2) immunophenotyping of malignant cells in the peripheral blood or bone marrow, (3) malignant cell count in bone marrow aspirates, peripheral blood smear or trephine biopsy, (4) a clinically established biomarker and (5) organ or tumor manifestation (defined by any imaging modality, for example, computed tomography (CT) scan or ultrasound). In vivo response to treatment that was initialized at study entry was classified as response, SD or PD according to the response criteria defined in the study protocol. One participant with CLL (S039) and primary lymph node involvement was not consistently followed up with CT scans. However, physical examination revealed the complete disappearance of the initial nodal manifestation and was considered a response. Individual participants with AML (S002, S012 and S037) or lymphoma (S049) were not followed up with bone marrow diagnostics or CT scans because they had a rapidly progressive course leading to death. In these participants, the response was considered PD.

#### Validation cohort

Response assessment was extracted from the SAL registry database. Briefly, participants were considered responders if they had less than 10% blasts in post-treatment bone marrow aspirates or a complete response with regeneration of white blood cells above 1 × 10^12^ m^−^^3^ and platelets above 1 × 10^14^ m^−^^3^. Participants were considered non-responders if the number of blasts in the bone marrow dropped by less than 50% or if they had disease progression. Overall survival and EFS were also obtained from the SAL registry database. For EFS, death, relapse and primary refractory disease were considered events.

### Genetic annotation of the validation cohort

Samples from the validation cohort were sequenced using the Twist Human Core Exome capture kit and a NovaSeq 6000 (Illumina). Raw reads from each read group were aligned against the GRCh37 genome (version hs37d5) using BWA mem (version 0.7.15) with option ‘-T 0’. The resulting BAM files were merged, and duplicates were marked using SamBamba markdup (version 0.6.5) with options ‘-t 1 -l 0–hash-table-size=2000000–overflow-list-size=1000000–io-buffer-size=64’. These alignments were generated using the Roddy alignment workflow plugin^22^ (version 1.2.73-204) in DKFZ OTP^23^. For small variant calling, a no-control strategy was used, which involved calling variants from the samples and removing common single-nucleotide polymorphisms and recurrent artifacts using variant frequency information from public and local control sample pools. The remaining variants, which contained somatic and rare germline variants, were used for downstream analysis. BCFtools mpileup (version 1.9) with options ‘-EI -q 30 -O u–ignore-RG–ff UNMAP,SECONDARY,QCFAIL,DUP,SUPPLEMENTARY -d 9999 -a AD -x’ was used to call the single-nucleotide variants, and Platypus (version 0.8.1.1) with options ‘–genIndels 1–genSNPs 1–bufferSize 100000–maxReads 5000000–minFlank 0’ was used to call the insertions/deletions from the merged BAM files. Furthermore, the variants were annotated with gencode v19 using ANNOVAR. Genomic region-based annotations and variant frequency information from 1,000 genomes, gnomAD (version 2.1) and local control databases were added using custom scripts. A confidence score was also added based on the region-based annotation, as previously described^24^. The local control contained variant frequency data from 4,879 whole-genome sequencing (WGS) and 1,198 whole-exome sequencing (WES) samples analyzed with the same workflows. Variants with a confidence score less than 8, a minor allele frequency above 0.01 in 1,000 genomes or 0.001 in gnomAD (WGS or WES) or with a frequency above 0.01 in the local control (WGS or WES) were annotated as common single-nucleotide polymorphisms or artifacts and removed from the downstream analysis. The no-control option of the Roddy SNVCallingWorkflow plugin^25^ (version 2.1.1) and Roddy IndelCallingWorkflow plugin^25^ (version 3.1.1) was used for the calling, annotation and filtering described above. FiLT3r (version b44c21f) was used to call the ITD in the *FLT3* gene with default parameters, except the reference sequence was updated to exon 14 and 15 regions in the GRCh37 coordinates (13:28607897-28608566)^26^.

### ELN-2022 risk classification

Risk categories were assigned based on the genetic information obtained by WES as well as karyotype and gene fusion annotation obtained from the SAL registry database^4^.

### Statistics and reproducibility

The primary endpoint of this study was the rate of successfully completed ex vivo DRPs within 7 d. The time between a participant’s inclusion in the study and the availability of the ex vivo drug profiling results was calculated. As secondary endpoints, we analyzed (1) the association between ex vivo and in vivo drug response testing and (2) the prediction of treatment failure by ex vivo drug response testing calculated as described in detail below.

Clinical baseline variables and outcome variables were summarized descriptively. To quantify the ex vivo response of acquired tumor cells to a specific drug at a given concentration, we used the viability relative to the median of 16 solvent controls (DMSO), excluding those on the outer boundaries of the plates. Technical replicates of the same drug and concentration per participant sample were averaged. The area under the curve (AUC) based on the trapezoidal rule was used to summarize the ex vivo drug effect across five drug concentrations. Dose–response curve visualizations for exemplary drugs were built by fitting a five-parameter logistic model using the drm function from the drc package for R^27^. To compare means between two or more groups, a standard unpaired *t*-test or analysis of variance was used, respectively. Correlations were measured as Pearson correlation coefficients or Kendall rank correlation coefficients, as indicated in the text and figures.

To evaluate the association between in vivo and ex vivo response (secondary endpoint 1), we assessed the ex vivo sensitivity in different in vivo response groups for both individual drugs and pathway groups. To compare means between the in vivo response and PD groups according to their ex vivo sensitivity to individual drugs, standard two-sample *t*-tests were performed. Additionally, in vivo response groups were compared for differential sensitivity at the pathway level by averaging the AUC values for the drugs belonging to the same pathway per participant sample.

To perform a covariate selection in the cohort of participants who received chemotherapy, we fitted an elastic net regularized logistic regression model with binary endpoint R versus PD (glmnet R package). Included covariates were ex vivo sensitivity (AUC) to all chemotherapeutics considered in the ex vivo drug testing. Only participants for whom both drug panels (myeloid and lymphoid) were available were considered in this model. The elastic net mixing parameter *α* (=0.3) was chosen to minimize overfitting while maintaining variable selection. To account for the small sample size, model selection was performed using only threefold cross-validation. AUROC was used as the model selection criterion. In total, we trained 1,000 models to check for the stability of results. For each covariate, median overall estimated coefficients (log (OR)) were computed. Median AUROC for each covariate was computed based on cross-validation AUROC of all models with a corresponding OR ≠ 1.

To predict treatment failure by ex vivo DRP in the SMARTrial cohort (secondary endpoint 2), we calculated the EFS and considered disease progression, change of treatment or death as treatment failure (event). EFS is defined as time from start of the scheduled treatment to PD, change of treatment or death. Participants without an event were censored at the date of last response and treatment assessment. To assess the value of ex vivo responses to individual drugs as predictors of EFS, univariate Cox proportional hazard regression modeling was performed using the R package ‘survival’. For visualization purposes, optimal cut points of drug responses were calculated using maximally selected log-rank statistics (maxstat R package). These cut points were used to split participants into two subcohorts and to plot their EFS with the Kaplan–Meier method. The median observation time from study inclusion was calculated by the reverse Kaplan–Meier estimate.

Because of the exploratory nature of the analyses in the SMARTrial cohort, *P* values here are reported without adjustment for multiple testing, and a *P* value of <0.05 was considered significant. For the validation cohort, *P* values were adjusted by the Benjamini–Hochberg method, and associations that passed a 10% false discovery rate cutoff (adjusted *P* value of <0.1) were reported as significant associations. All statistical analyses were performed in R version 4.1.3 (R Foundation for Statistical Computing). The same data-processing steps and statistical tests were performed for both the SMARTrial and the validation cohorts. For the SMARTrial, sample size was based on the approach described in Li and Fine for testing sensitivity^28^, and details are described in Supplementary Information. Data distribution was assumed to be normal, but this was not formally tested. Participants and samples were not distributed into groups, and randomization was therefore not performed. Data collection and analysis were not performed blind to the conditions of the experiments. Further information on research design is available in the Nature Research Reporting Summary linked to this article.

### Reporting summary

Further information on research design is available in the Nature Portfolio Reporting Summary linked to this article.

### Supplementary information

Supplementary Information Supplementary Tables 1–7 and SMARTrial study protocol.

Reporting Summary

### Source data

Source Data Fig. 1 Statistical source data and graphic for visual abstract.

Source Data Fig. 2 Statistical source data.

Source Data Fig. 3 Statistical source data.

Source Data Fig. 4 Statistical source data.

Source Data Extended Data Fig. 1 Statistical source data.

Source Data Extended Data Fig. 2 Statistical source data.

Source Data Extended Data Fig. 3 Statistical source data.

Source Data Extended Data Fig. 4 Statistical source data.

Source Data Extended Data Fig. 5 Statistical source data.

Source Data Extended Data Fig. 6 Statistical source data.

Source Data Extended Data Fig. 7 Statistical source data and radiological images.

Source Data Extended Data Fig. 8 Statistical source data.

Source Data Extended Data Fig. 9 Statistical source data.

Source Data Extended Data Fig. 10 Statistical source data.

## Data Availability

All data reported in this paper are publicly available at https://github.com/PeterBruch/SMARTrial. Additionally, the SMARTrial data can be explored through our interactive web application at http://mozi.embl.de/public/SMARTrial/. WES data obtained for the AML validation cohort are available at the European Genome–Phenome Archive under accession number EGAS00001007223. Source data are provided with this paper.

## Code availability

The computational code, in the form of Rmarkdown documents, for reproducing all major figures and results reported in this article is provided at GitHub (https://github.com/PeterBruch/SMARTrial) under GNU General Public License v.3.0.
