# Supplementary material for: Ex vivo drug response profiling for response and outcome prediction in hematologic malignancies: the prospective non-interventional SMARTrial
Source: Nat Cancer. 2023 Oct 2;4(12):1648–59. doi: 10.1038/s43018-023-00645-5 (PMC10733146; doi:10.1038/s43018-023-00645-5)
Supplement: Supplementary file 1 — Supplementary Tables 1–7 and SMARTrial study protocol. [file 43018_2023_645_MOESM1_ESM.pdf]

nature cancer

Article

https://doi.org/10.1038/s43018-023-00645-5

Ex vivo drug response profiling for response and outcome prediction in hematologic malignancies: the prospective non-interventional SMARTrial

In the format provided by the authors and unedited

Table of contents

**Supplementary Tables**	Page
Supplementary Table S1	3
Supplementary Table S2	12
Supplementary Table S3	12
Supplementary Table S4	19
Supplementary Table S5	21
Supplementary Table S6	25
Supplementary Table S7	30
**SMARTrial study protocol**	**31**

Supplementary tables

Supplementary Table S1: Detailed patient characteristics

Patient ID	Diagnosis	Age	Sex	Type of sample	Tumor infiltration	Number of previous treatments	Prescribed treatment	Response defining parameter	Response according to study protocol	Death	Event-free survival time
S002	AML	71	Female	PB	88	0	Induction (cytarabine and daunorubicin)	Immunophenotyping	PD	1	18
S003	CLL	69	Female	KM	86	2	Venetoclax	Peripheral blood smear	R	1	378
S005	BL	31	Male	LN	70	1	R-DHAP + autoHCT	Organ/tumor manifestation	PD	0	48
S006	AML	78	Female	PB	83	0	Induction (cytarabine and daunorubicin)	BM aspiration/ BM biopsy	R	1	40
S007	AML	59	Female	PB	93	0	Induction (cytarabine and daunorubicin)	BM aspiration/ BM biopsy	R	0	29
S008	T-PLL	56	Male	PB	96	0	Alemtuzumab+FCM	Blood counts	R	1	160
S009	AML	63	Male	KM	82	0	Induction (cytarabine and daunorubicin)	BM aspiration/ BM biopsy	R	0	977

S011	AML	79	Male	PB	92	0	Cytarabine pretreatment + Vidaza	BM aspiration/ BM biopsy	R	1	225
S012	AML	81	Male	KM	59	0	Vidaza	BM aspiration/ BM biopsy	PD	1	3
S013	DLBCL	41	Female	PB	81	2	Ibrutinib + Rituximab	Blood counts	R	0	211
S014	CLL	69	Male	PB	71	2	Ibrutinib	Peripheral blood smear	R	1	406
S015	CLL	78	Male	PB	88	0	Ibrutinib	Peripheral blood smear	R	1	434
S016	B-PLL	77	Female	PB	84	0	Rituximab-Bendamustine	Blood counts	R	1	469
S017	AML	67	Male	KM	55	0	Induction (cytarabine and daunorubicin)	BM aspiration/ BM biopsy	R	1	36
S019	AML	51	Female	KM	60	0	Induction (cytarabine and daunorubicin)+ Midostaurin	BM aspiration/ BM biopsy	R	0	28
S020	T-PLL	80	Male	PB	93	0	Cyclophosphamide + Alemtuzumab	Peripheral blood smear	na	1	na
S021	AML	71	Female	PB	87	0	Induction (cytarabine and daunorubicin)	BM aspiration/ BM biopsy	R	1	606

S022	FL	71	Male	LN	77	1	Bendamustin	Organ/tumor manifestation	R	0	84
S023	CLL	50	Female	PB	77	4	Ibrutinib	Blood counts	R	0	775
S024	AML	64	Male	PB	80	0	Induction (cytarabine and daunorubicin)	BM aspiration/ BM biopsy	na	1	na
S025	AML	24	Male	PB	88	0	Induction (cytarabine and daunorubicin)	BM aspiration/ BM biopsy	R	0	34
S027	AML	64	Male	PB	79	0	Induction (cytarabine and daunorubicin)	BM aspiration/ BM biopsy	R	0	220
S028	CLL	53	Male	PB	89	1	Ibrutinib	Peripheral blood smear	R	0	974
S029	FL	50	Male	LN	52	2	Idelalisib	Organ/tumor manifestation	R	0	813
S030	CLL	78	Male	PB	83	1	Ibrutinib	Peripheral blood smear	na	0	na
S031	CLL	79	Male	LN	85	0	Rituximab-Benda-mustine	Organ/tumor manifestation	R	0	792
S032	CLL	57	Female	PB	97	0	Ibrutinib	Blood counts	na	0	na

S033	CLL	86	Female	PB	82	3	Venetoclax-Rituximab	Blood counts	R	0	783
S034	CLL	70	Female	PB	84	1	Ibrutinib	Organ/tumor manifestation	na	0	na
S035	CLL	75	Male	PB	90	2	Venetoclax-Rituximab	Peripheral blood smear	R	0	882
S037	AML	82	Male	PB	80	0	Vidaza	BM aspiration/ BM biopsy	PD	1	28
S038	CLL	50	Male	PB	63	0	Ibrutinib	Peripheral blood smear	R	0	590
S039	CLL	73	Male	LN	88	1	Venetoclax-Rituximab	Organ/tumor manifestation	R	0	767
S040	T-PLL	74	Male	PB	76	0	Alemtuzumab	Peripheral blood smear	R	1	451
S041	MCL	64	Male	PB	89	0	R-CHOP/R-DHAP +au-toSCT	Blood counts	R	1	441
S042	AML	78	Male	PB	57	0	Palbociclib	Peripheral blood smear	PD	1	39
S043	CLL	78	Female	PB	88	0	Rituximab-Benda-mustine	Blood counts	R	0	624
S044	AML	74	Male	PB	75	0	Induction (cytarabine and daunorubicin)	Peripheral blood smear	R	1	34

S045	AML	81	Female	PB	90	0	Cytarabine	BM aspiration/ BM biopsy	na	1	na
S046	CLL	79	Female	PB	83	2	Rituximab-Idelalisib	Blood counts	R	0	682
S047	T-PLL	81	Male	PB	92	0	Alemtuzumab	Peripheral blood smear	R	1	426
S048	ALL	20	Male	PB	94	0	GMALL protocol	BM aspiration/ BM biopsy	R	0	645
S049	DLBCL	63	Female	LN	88	3	Rituximab-Oxaliplatin	Organ/tumor manifestation	PD	1	7
S050	AML	63	Female	KM	75	0	Induction (cytarabine and daunorubicin)	Peripheral blood smear	SD	1	13
S052	CLL	64	Female	PB	93	1	Ibrutinib	Organ/tumor manifestation	R	0	550
S053	MCL	51	Male	PB	73	0	R-DHAP + autoSCT	Organ/tumor manifestation	R	0	193
S054	AML	66	Female	PB	54	0	Induction (cytarabine and daunorubicin)	BM aspiration/ BM biopsy	R	0	32
S055	AML	56	Male	PB	97	0	Induction (cytarabine and daunorubicin)	BM aspiration/ BM biopsy	R	0	550

S056	AML	67	Female	PB	87	1	Cytarabine/Bortezomib/Mitoxantron+Quizartinib + alloHCT	Peripheral blood smear	R	1	56
S058	T-NHL	51	Female	LN	90	3	Rituximab-Bendamustine	Organ/tumor manifestation	PD	1	35
S060	AML	46	Female	PB	97	0	Induction (cytarabine and daunorubicin)	BM aspiration/ BM biopsy	R	0	550
S061	AML	78	Female	PB	89	0	Induction (cytarabine and daunorubicin)	BM aspiration/ BM biopsy	SD	1	13
S062	AML	50	Male	PB	60	0	Induction (cytarabine and daunorubicin) + Midostaurin	BM aspiration/ BM biopsy	R	0	82
S063	MCL	77	Female	LN	58	0	Rituximab-Bendamustine	Organ/tumor manifestation	R	0	216
S064	AML	58	Female	KM	61	0	Induction (cytarabine and daunorubicin)	BM aspiration/ BM biopsy	SD	0	17
S065	CLL	72	Male	PB	96	1	Venetoclax-Rituximab	Blood counts	R	0	550
S066	CLL	85	Male	PB	85	1	Venetoclax-Rituximab	Blood counts	R	1	257

S067	CLL	68	Female	PB	99	0	Ibrutinib	Blood counts	R	0	547
S068	MCL	51	Male	PB	94	0	Ibrutinib + R-CHOP/R-DHAP	Peripheral blood smear	R	0	548
S069	CLL	91	Male	PB	92	1	Ibrutinib	Peripheral blood smear	SD	0	548
S070	CLL	67	Female	PB	89	0	Ibrutinib	Peripheral blood smear	R	0	548
S071	AML	76	Male	KM	59	0	Induction (cytarabine and daunorubicin)	BM aspiration/ BM biopsy	R	1	129
S072	AML	70	Male	KM	68	0	Induction (liposomal cy-tarabine and dauno-rubicin)	BM aspiration/ BM biopsy	R	1	82
S074	AML	69	Male	PB	97	0	Induction (cytarabine and daunorubicin) after cytarabine prephase	BM aspiration/ BM biopsy	SD	0	16
S075	AML	23	Female	PB	90	0	Induction (cytarabine and daunorubicin)	BM aspiration/ BM biopsy	SD	1	16
S076	DLBCL	74	Female	KM	87	0	R-CHOP	Peripheral blood smear	R	1	274
S077	MCL	67	Male	PB	54	4	Venetoclax	Blood counts	na	1	na

S078	CLL	57	Female	PB	88	0	Ibrutinib	Peripheral blood smear	R	0	366
S079	AML	27	Female	PB	78	0	Induction (cytarabine and daunorubicin)+ Gemtuzumab Ozogami-cin	BM aspiration/ BM biopsy	R	0	182
S080	CLL	73	Male	PB	86	2	Ibrutinib	Peripheral blood smear	na	1	na
S081	AML	78	Male	PB	70	0	Venetoclax-Vidaza	BM aspiration/ BM biopsy	R	1	143
S082	DLBCL	77	Female	PB	74	2	R-miniCHOP	Organ/tumor manifestation	PD	1	28
S083	AML	58	Male	KM	53	2	Cytarabine	BM aspiration/ BM biopsy	na	1	na
S085	AML	79	Male	KM	74	0	Venetoclax-Vidaza	BM aspiration/ BM biopsy	R	0	365
S086	CLL	81	Male	PB	96	3	Venetoclax-Rituximab	Peripheral blood smear	R	0	365
S087	FL	78	Male	LN	68	0	R-miniCHOP	Organ/tumor manifestation	R	0	177

S088	ALL	23	Male	PB	89	0	GMALL protocol	BM aspiration/ BM biopsy	R	1	348
S089	AML	65	Male	PB	83	0	Induction (cytarabine and daunorubicin) + Midostaurin	BM aspiration/ BM biopsy	R	0	25
S090	CLL	48	Male	PB	95	0	Venetoclax + Obinutuzumab	Peripheral blood smear	R	0	365
S091	AML	36	Male	PB	84	0	Induction (cytarabine and daunorubicin)	BM aspiration/ BM biopsy	R	0	19

Note: Induction treatment (as specified in prescribed treatment) and subsequent consolidation treatment was considered as one treatment line.
AML, acute myeloid leukemia; ALL, acute lymphocytic leukemia; DLBCL, diffuse large B-cell lymphoma; CLL, chronic lymphocytic leukemia; B-PLL, B-cell prolymphocytic leukemia; T-PLL, T-cell prolymphocytic leukemia; NOS, not otherwise specified; PB: peripheral blood; BM: bone marrow; LN: lymph node; autoHCT: autologous stem cell transplantation; alloHCT: allogeneic stem cell transplantation; R-DHAP: rituximab, high dose cytarabine, cisplatin, dexamethasone; R-CHOP: rituximab, cyclophosphamide, doxorubicin, vincristine, prednisolone; FCM: fludarabine, cyclophosphamide, mitoxantrone; GMALL protocol: treatment according to protocols of the German Multicenter Study Group for Adult ALL; R: clinical response; SD: stable disease; PD: progressive disease, na: not available

Supplementary Table S2: Median time from diagnosis of relapse / treatment indication to treatment initiation

Diagnosis	Median	Range	Number of cases
AML	2.5	0 - 15	34
CLL	21.0	0 - 144	25
MCL	8.0	0 - 49	5
DLBCL	14.5	2 - 27	4
T-PLL	2.0	1 - 6	4
FL	32.0	22 - 82	3
ALL	5.0	0 - 10	2
B-PLL	6.0	6 - 6	1
BL	8.0	8 - 8	1
T-NHL	22.0	22 - 22	1

Supplementary Table S3: Drugs included in the SMARTrial drug screen

Compound	Simplified drug class/ targeted pathway	Main targets/ mode of action	Supplier
10058-F4	Cell cycle	MYC	Sigma-Aldrich
8-Azaguanin	Chemotherapy	Purine analogue	Selleck Chemicals
Abemaciclib (LY2835219)	CDK	CDK 4+6	Selleck Chemicals
Acalabrutinib (ACP-196)	BCR	BTK	Selleck Chemicals
AG-120 (Ivosidenib)	IDH	IDH 1	Selleck Chemicals
AG-221 (Enasidinib)	IDH	IDH 2	Selleck Chemicals
AGI-6780	IDH	IDH 2	Selleck Chemicals
Azacytidine (Vidaza)	Chemotherapy	DNA Methylation	Biomol
AZD7762	DDR	CHEK1, CHEK2	Selleck Chemicals
Barasertib	Cell cycle	AURKB	Selleck Chemicals
BAY 1436032	IDH	mIDH 1	BioCat
Bendamustine	Chemotherapy	DNA	Selleck Chemicals

BEZ235 (Dactolisib)	PI3K/AKT/mTOR	PIK3CA, PIK3CG, PIK3CG, MTOR	Selleck Chemicals
BI6727 (Volasertib)	PLK	PLK1	Selleck Chemicals
Birinapant	Apoptosis	BIRC2	Selleck Chemicals
Bortezomib	Proteasome	PSMB5	Selleck Chemicals
BRD73954	Histone deacetylase	HDAC6, HDAC8	Selleck Chemicals
C646	Histone acetyltransferase	EP300	Selleck Chemicals
Carfilzomib	Proteasome	PSMB5	Selleck Chemicals
CAY10603	Histone deacetylase	HDAC6	Selleck Chemicals
Ceritinib	ALK	ALK	Selleck Chemicals
Chlorambucil	Chemotherapy	Alkylation	Selleck Chemicals
Cisplatin*	Chemotherapy	DNA	Selleck Chemicals
Cladribine	Chemotherapy	Purine analogue	Selleck Chemicals
Clofarabine	Chemotherapy	Purine analogue	Selleck Chemicals
CPI-169	Histone methyltransferase	EZH2	Selleck Chemicals
Crenolanib	FLT3	FLT3	Selleck Chemicals
Crizotinib	HGF	MET, ALK	Selleck Chemicals
Cyclophosphamide[§]	Chemotherapy	Alkylation	Selleck Chemicals
Cytarabine	Chemotherapy	DNA	Sigma-Aldrich

Dasatinib	ABL (BCR)	ABL1, SRC, KIT	Selleck Chemicals
Daunorubicin	Chemotherapy	DNA, TOP2A, TOP2B	Selleck Chemicals
Decitabine (Dacogen)	Chemotherapy	DNA Methylation	Selleck Chemicals
Dinaciclib	CDK	CDK2, CDK5, CDK1, CDK9	Selleck Chemicals
Doxorubicin	Chemotherapy	TOP2A, TOP2B	Selleck Chemicals
Duvelisib	PI3K/AKT/mTOR	PI3KCD, PIK3CG	Selleck Chemicals
Entospletinib	BCR	SYK	Selleck Chemicals
EPZ-5676	Histone methyltransferase	DOT1L	Selleck Chemicals
EPZ-6438	Histone methyltransferase	EZH2	Selleck Chemicals
ERK5-IN-1	MAPK	MAPK7	Selleck Chemicals
Etoposide	Chemotherapy	TOP2A, TOP2B	Selleck Chemicals
Everolimus	PI3K/AKT/mTOR	MTOR	Selleck Chemicals
EVP4593	TNF/NFKB	TNF, NFKB1, NFKB2	Selleck Chemicals
Filgotinib	JAK/STAT	JAK1	Selleck Chemicals
Flavopiridol	CDK	CDK1, CDK2, CDK4, CDK5, CDK6, CDK9	Selleck Chemicals
Fludarabine	Chemotherapy	POLA1, DNA	Selleck Chemicals
Ganetespib	Stress response	HSP90AA1, HSP90AB1	Selleck Chemicals
Gilteritinib	FLT3	FLT3	Selleck Chemicals

I-BET-762	Bromodomain	BRD2, BRD3, BRD4	Selleck Chemicals
Ibrutinib	BCR	BTK	Selleck Chemicals
Idarubicin	Chemotherapy	Topoisomerase II	Selleck Chemicals
Idasanutlin (RS7388)	DDR	MDM2	Selleck Chemicals
Idelalisib	PI3K/AKT/mTOR	PIK3CD	Selleck Chemicals
Imatinib	ABL (BCR)	BCR/ABL, ABL1, KIT, PDGFRA, PDGFRB	Selleck Chemicals
Iniparib	DDR	PARP1	Selleck Chemicals
IRAK 4 Compound 26	TLR	IRAK4	Merck Chemicals
IRAK-1/4 INHIBITOR I	TLR	IRAK1, IRAK4	Sigma-Aldrich
Ixazomib	Proteasome	Proteasome	Selleck Chemicals
JAK3 Inhibitor I	JAK/STAT	JAK3	Merck Chemicals
Lenalidomide	TNF/NFKB	TNFSF11	Selleck Chemicals
LY3039478	Notch	Notch-1	Selleck Chemicals
MI-773	DDR	MDM2	Selleck Chemicals
Midostaurin hydrate	PKC	PRKCA, PRKCG, PRKCB, KDR, SYK	Sigma-Aldrich
Mitoxantrone	Chemotherapy	TOP2A, DNA	Sigma-Aldrich
MLN-120B	TNF/NFKB	IKBKB	Sigma-Aldrich
Navitoclax	Apoptosis	BCL2, BCL2L1, BCL2L2	Selleck Chemicals
Nelarabine	Chemotherapy	Purine analogue	Selleck Chemicals

Nilotinib	ABL (BCR)	BCR/ABL	Sigma-Aldrich
NSC 652287	DDR	MDM2, DNA	Selleck Chemicals
Nutlin-3a	DDR	MDM2	Selleck Chemicals
Obatoclax mesylate	Apoptosis	BCL2, BCL2L1, MCL1	Selleck Chemicals
Olaparib	DDR	PARP1, PARP2	Selleck Chemicals
Onalespib	Stress response	HSP90AA1, HSP90AB1	Selleck Chemicals
ONO-4059	BCR	BTK	Selleck Chemicals
OTX015	Bromodomain	BRD2, BRD3, BRD4	Selleck Chemicals
Pacritinib	JAK/STAT	JAK 2	Selleck Chemicals
Palbociclib	CDK	CDK4, CDK6	Selleck Chemicals
Panobinostat	Histone deacetylase	HDAC1, HDAC2, HDAC3, HDAC4, HDAC5, HDAC6, HDAC7, HDAC8, HDAC9, HDAC10, HDAC11	Selleck Chemicals
Pentostatin	Chemotherapy	ADA	Sigma-Aldrich
Pomalidomide	TNF/NFKB	TNF	Selleck Chemicals
Pralatrexate	Chemotherapy	DHFR	Selleck Chemicals
Quizartinib	FLT3	FLT3	Selleck Chemicals
Resveratrol	Promiscuous	SIRT1, SIRT2, PTGS1, PTGS2, ALOX15, ALOX5	Selleck Chemicals
Ribociclib	CDK	CDK4, CDK6	Selleck Chemicals
Rigosertib	PLK	PLK1	Selleck Chemicals

Romidepsin	Histone deacetylase	HDAC1, HDAC2	Selleck Chemicals
Roscovitine	CDK	CDK1, CDK2, CDK5	Selleck Chemicals
Ruxolitinib	JAK/STAT	JAK1, JAK2	Selleck Chemicals
SCH772984	MAPK	MAPK3, MAPK1	Selleck Chemicals
Selinexor	Nuclear export	XPO1	Selleck Chemicals
SGC 0946	Histone methyltransferase	DOT1L	Selleck Chemicals
Sorafenib	MAPK	RAF1, BRAF, KDR	Selleck Chemicals
Temsirolimus	PI3K/AKT/mTOR	MTOR	Selleck Chemicals
Thalidomide	TNF/NFKB	TNF	Selleck Chemicals
Thioguanin	Chemotherapy	Purine analogue	Selleck Chemicals
TMP269	Histone deacetylase	HDAC4, HDAC5, HDAC7, HDAC9	Selleck Chemicals
Tofacitinib	JAK/STAT	JAK1, JAK3	Selleck Chemicals
Tozasertib	Cell cycle	AURKA, AURKB	Selleck Chemicals
Trametinib	MAPK	MAP2K1, MAP2K2	Selleck Chemicals
UMI-77	Apoptosis	MCL1	Selleck Chemicals
Vemurafenib	MAPK	BRAF(V600E)	Selleck Chemicals
Venetoclax	Apoptosis	BCL2	Selleck Chemicals
Vincristine	Chemotherapy	Vinca alkaloid	Selleck Chemicals

Vindesine	Chemotherapy	Vinca alkaloid	Sigma-Aldrich
Vismodegib	Hedgehog	SMO	Selleck Chemicals
Vorinostat	Histone deacetylase	HDAC1, HDAC3	Selleck Chemicals

* Due to incompatibility of cisplatin with DMSO, this drug was excluded from analyses.

§ Due to the *in vitro* inefficacy of the prodrug, this drug was excluded from analyses.

Supplementary Table S4: Results of the elastic net logistic regression model showing all included covariates (corresponding to Figure 3D)

Covariate (drug)	Median OR	Selected proportion	Median AUROC
Vincristine	0.96	1	0.85
Idarubicin	0.97	0.99	0.85
Vindesine	0.97	0.99	0.85
Mitoxantrone	0.97	0.97	0.85
Cladribine	0.97	0.88	0.84
Fludarabine	0.98	0.53	0.83
Azaguanin	0.97	0.45	0.84
Etoposide	0.97	0.45	0.84
Thioguanin	0.93	0.39	0.84
Pentostatin	1.12	0.26	0.84
Bendamustine	0.94	0.25	0.85
Pralatrexate	0.92	0.25	0.85
Nelarabine	1.01	0.15	0.86
Clofarabine	1.14	0.15	0.86
Daunorubicin	1.13	0.14	0.87
Cytarabine	1.07	0.12	0.87
Doxorubicin	1.03	0.11	0.87
Chlorambucil	1.01	0.11	0.88
Vidaza	1.01	0.10	0.87
Decitabine	1.07	0.01	0.88
(Intercept)	1.00	0	0.50

Results of the elastic net logistic regression model with the binary endpoint response vs progressive disease (R: n=33, PD: n=5, only patients for whom both drug panels were available were considered). *Ex vivo* drug viability (AUC) to all chemotherapeutic agents of the *ex vivo* drug response profiling were

included. Model fitting was repeated 1000 times to check for stability of results. For each covariate, median overall estimated coefficients (log odds ratio (OR)) were computed. The median OR presented here relates to a change of *ex vivo* drug viability of 10%. The covariates are ordered by the frequency of all corresponding estimated ORs that indicate an association between the *ex vivo* viability and the binary endpoint (i.e., OR≠1) considering all 1000 models (selected proportion). Median Area under the ROC curve (AUROC) for each covariate was computed based on cross-validation AUROC of all models with a corresponding OR≠1.

Supplementary Table S5: Patient characteristics Validation cohort

Patien-tID	Age	Sex	Treatment	Tumor cell infiltration	Diagnosis	In vivo re-sponse group	ELN-22 risk group
AML_1	57	male	Cytarabin + Daunorubicin	70,5	AML	Non-Responder	adverse
AML_2	59	female	Cytarabin + Daunorubicin	71	AML	Non-Responder	adverse
AML_3	67	male	Cytarabin + Daunorubicin	91	AML	Non-Responder	adverse
AML_4	45	male	Cytarabin + Daunorubicin	60,5	AML	Non-Responder	adverse
AML_5	71	female	Cytarabin + Daunorubicin	80	AML	Non-Responder	adverse
AML_6	63	male	Cytarabin + Daunorubicin	88	AML	Non-Responder	adverse
AML_7	55	male	Cytarabin + Daunorubicin	65	AML	Non-Responder	adverse
AML_8	18	male	Cytarabin + Daunorubicin	90	AML	Non-Responder	adverse
AML_9	59	male	Cytarabin + Daunorubicin	67	AML	Non-Responder	adverse
AML_10	69	female	Cytarabin + Daunorubicin	80	AML	Non-Responder	adverse
AML_11	64	male	Cytarabin + Daunorubicin	85	AML	Non-Responder	adverse
AML_12	31	female	Cytarabin + Daunorubicin	69	AML	Non-Responder	adverse
AML_13	64	male	Cytarabin + Daunorubicin	77	AML	Non-Responder	adverse
AML_14	59	male	Cytarabin + Daunorubicin	87	AML	Non-Responder	adverse
AML_15	62	male	Cytarabin + Daunorubicin	60	AML	Non-Responder	adverse
AML_16	65	male	Cytarabin + Daunorubicin	60	AML	Non-Responder	adverse
AML_17	75	male	Cytarabin + Daunorubicin	50,5	AML	Non-Responder	adverse
AML_18	54	male	Cytarabin + Daunorubicin	70	AML	Non-Responder	adverse
AML_19	61	female	Cytarabin + Daunorubicin	58	AML	Non-Responder	adverse
AML_20	81	female	Cytarabin + Daunorubicin	72	AML	Non-Responder	adverse
AML_21	58	female	Cytarabin + Daunorubicin	92	AML	Non-Responder	adverse
AML_22	59	male	Cytarabin + Daunorubicin	99	AML	Non-Responder	adverse
AML_23	68	male	Cytarabin + Daunorubicin	64	AML	Non-Responder	adverse

AML_24	61	male	Cytarabin + Daunorubicin	80	AML	Non-Responder	adverse
AML_25	33	male	Cytarabin + Daunorubicin	65	AML	Non-Responder	adverse
AML_26	65	male	Cytarabin + Daunorubicin	93	AML	Non-Responder	adverse
AML_27	74	male	Cytarabin + Daunorubicin	60	AML	Non-Responder	adverse
AML_28	42	male	Cytarabin + Daunorubicin	80	AML	Non-Responder	adverse
AML_29	64	female	Cytarabin + Daunorubicin	65,5	AML	Non-Responder	favorable
AML_30	39	female	Cytarabin + Daunorubicin	84	AML	Non-Responder	favorable
AML_31	47	male	Cytarabin + Daunorubicin	95	AML	Non-Responder	favorable
AML_32	49	female	Cytarabin + Daunorubicin	90	AML	Non-Responder	favorable
AML_33	63	male	Cytarabin + Daunorubicin	90	AML	Non-Responder	favorable
AML_34	22	female	Cytarabin + Daunorubicin	81	AML	Non-Responder	favorable
AML_35	23	male	Cytarabin + Daunorubicin	89	AML	Non-Responder	intermediate
AML_36	61	female	Cytarabin + Daunorubicin	91	AML	Non-Responder	intermediate
AML_37	26	male	Cytarabin + Daunorubicin	55	AML	Non-Responder	intermediate
AML_38	65	male	Cytarabin + Daunorubicin	90	AML	Non-Responder	intermediate
AML_39	62	female	Cytarabin + Daunorubicin	61	AML	Non-Responder	intermediate
AML_40	63	female	Cytarabin + Daunorubicin	85	AML	Non-Responder	intermediate
AML_41	69	female	Cytarabin + Daunorubicin	60	AML	Non-Responder	intermediate
AML_42	42	female	Cytarabin + Daunorubicin	88	AML	Non-Responder	intermediate
AML_43	54	female	Cytarabin + Daunorubicin	67	AML	Non-Responder	intermediate
AML_44	35	male	Cytarabin + Daunorubicin	72	AML	Non-Responder	intermediate
AML_45	51	male	Cytarabin + Daunorubicin	90	AML	Non-Responder	intermediate
AML_46	62	female	Cytarabin + Daunorubicin	64	AML	Non-Responder	intermediate
AML_47	58	female	Cytarabin + Daunorubicin	59	AML	Non-Responder	intermediate
AML_48	69	male	Cytarabin + Daunorubicin	90	AML	Non-Responder	intermediate

AML_49	72	female	Cytarabin + Daunorubicin	80	AML	Responder	adverse
AML_50	64	female	Cytarabin + Daunorubicin	75,5	AML	Responder	adverse
AML_51	62	male	Cytarabin + Daunorubicin	59	AML	Responder	adverse
AML_52	71	female	Cytarabin + Daunorubicin	89,5	AML	Responder	adverse
AML_53	72	female	Cytarabin + Daunorubicin	90	AML	Responder	adverse
AML_54	73	female	Cytarabin + Daunorubicin	67	AML	Responder	adverse
AML_55	25	female	Cytarabin + Daunorubicin	95	AML	Responder	adverse
AML_56	51	male	Cytarabin + Daunorubicin	51	AML	Responder	adverse
AML_57	62	female	Cytarabin + Daunorubicin	61	AML	Responder	favorable
AML_58	57	female	Cytarabin + Daunorubicin	67	AML	Responder	favorable
AML_59	31	male	Cytarabin + Daunorubicin	50	AML	Responder	favorable
AML_60	50	female	Cytarabin + Daunorubicin	73,8	AML	Responder	favorable
AML_61	69	male	Cytarabin + Daunorubicin	69	AML	Responder	favorable
AML_62	59	female	Cytarabin + Daunorubicin	67	AML	Responder	favorable
AML_63	55	male	Cytarabin + Daunorubicin	51	AML	Responder	favorable
AML_64	33	female	Cytarabin + Daunorubicin	80	AML	Responder	favorable
AML_65	24	female	Cytarabin + Daunorubicin	61	AML	Responder	favorable
AML_66	67	female	Cytarabin + Daunorubicin	82	AML	Responder	intermediate
AML_67	50	male	Cytarabin + Daunorubicin	90	AML	Responder	intermediate
AML_68	63	female	Cytarabin + Daunorubicin	66	AML	Responder	intermediate
AML_69	84	female	Cytarabin + Daunorubicin	55	AML	Responder	intermediate
AML_70	74	female	Cytarabin + Daunorubicin	90	AML	Responder	intermediate
AML_71	66	female	Cytarabin + Daunorubicin	53	AML	Responder	intermediate
AML_72	58	female	Cytarabin + Daunorubicin	90,5	AML	Responder	intermediate
AML_73	40	male	Cytarabin + Daunorubicin	86	AML	Responder	intermediate

AML_74	35	female	Cytarabin + Daunorubicin	97	AML	Responder	intermediate
AML_75	51	male	Cytarabin + Daunorubicin	95	AML	Responder	intermediate
AML_76	58	female	Cytarabin + Daunorubicin	50	AML	Responder	intermediate
AML_77	23	female	Cytarabin + Daunorubicin	80	AML	Responder	intermediate
AML_78	65	male	Cytarabin + Daunorubicin	70	AML	Responder	intermediate
AML_79	53	male	Cytarabin + Daunorubicin	74	AML	Responder	intermediate
AML_80	66	female	Cytarabin + Daunorubicin	97	AML	Responder	intermediate
AML_81	44	male	Cytarabin + Daunorubicin	87	AML	Responder	intermediate
AML_82	55	male	Cytarabin + Daunorubicin	90	AML	Responder	intermediate
AML_83	71	male	Cytarabin + Daunorubicin	76	AML	Responder	intermediate
AML_84	52	female	Cytarabin + Daunorubicin	80	AML	Responder	intermediate
AML_85	63	male	Cytarabin + Daunorubicin	55,5	AML	Responder	intermediate
AML_86	18	female	Cytarabin + Daunorubicin	91,5	AML	Responder	intermediate
AML_87	59	female	Cytarabin + Daunorubicin	71,5	AML	Responder	intermediate
AML_88	52	female	Cytarabin + Daunorubicin	71	AML	Responder	intermediate
AML_89	19	male	Cytarabin + Daunorubicin	94,5	AML	Responder	intermediate
AML_90	70	female	Cytarabin + Daunorubicin	90	AML	Responder	intermediate
AML_91	64	male	Cytarabin + Daunorubicin	87	AML	Responder	intermediate
AML_92	59	male	Cytarabin + Daunorubicin	90	AML	Responder	intermediate
AML_93	65	male	Cytarabin + Daunorubicin	70	AML	Responder	intermediate
AML_94	75	male	Cytarabin + Daunorubicin	72	AML	Responder	intermediate
AML_95	62	female	Cytarabin + Daunorubicin	75	AML	Responder	favorable

Supplementary Table S6: Drug concentrations used in the SMARTrial drug screen.

Drug	Concentration 1 [µM]	Concentration 2 [µM]	Concentration 3 [µM]	Concentration 4 [µM]	Concentration 5 [µM]
10058-F4	15	3	0.6	0.12	0.024
8-Azaguanin	20	4	0.8	0.16	0.032
Abemaciclib (LY2835219)	15	3	0.6	0.12	0.024
Acalabrutinib (ACP-196)	5	1	0.2	0.04	0.008
AG-120	15	3	0.6	0.12	0.024
AG-221 (Enasidinib)	15	3	0.6	0.12	0.024
AGI-6780	15	3	0.6	0.12	0.024
Azacytidine (Vidaza)	20	4	0.8	0.16	0.032
AZD7762	15	3	0.6	0.12	0.024
Barasertib	0.5	0.1	0.02	0.004	0.0008
BAY 1436032	15	3	0.6	0.12	0.024
Bendamustine	20	4	0.8	0.16	0.032
BEZ235	15	3	0.6	0.12	0.024
BI6727	15	3	0.6	0.12	0.024
Birinapant	0.5	0.1	0.02	0.004	0.0008
Bortezomib	5	1	0.2	0.04	0.008
BRD73954	15	3	0.6	0.12	0.024
C646	15	3	0.6	0.12	0.024
Carfilzomib	0.1	0.02	0.004	0.0008	0.00016
CAY10603	15	3	0.6	0.12	0.024

Ceritinib	15	3	0.6	0.12	0.024
Chlorambucil	20	4	0.8	0.16	0.032
Cisplatin	46.9	9.38	1.876	0.375	0.075
Cladribine	15	3	0.6	0.12	0.024
Clofarabine	15	3	0.6	0.12	0.024
CPI-169	15	3	0.6	0.12	0.024
Crenolanib	10	2	0.4	0.08	0.016
Crizotinib	15	3	0.6	0.12	0.024
Cyclophospha-mide	20	4	0.8	0.16	0.032
Cytarabine	20	4	0.8	0.16	0.032
Dasatinib	1	0.2	0.04	0.008	0.0016
Daunorubicin	1	0.2	0.04	0.008	0.0016
Decitabine (Dacogen)	15	3	0.6	0.12	0.024
Dinaciclib	1	0.2	0.04	0.008	0.0016
Doxorubicin	1	0.2	0.04	0.008	0.0016
Duvelisib	1	0.2	0.04	0.008	0.0016
Entospletinib	15	3	0.6	0.12	0.024
EPZ-5676	2	0.4	0.08	0.016	0.0032
EPZ-6438	15	3	0.6	0.12	0.024
ERK5-IN-1	15	3	0.6	0.12	0.024
Etoposide	20	4	0.8	0.16	0.032
Everolimus	2	0.4	0.08	0.016	0.0032

EVP4593	5	1	0.2	0.04	0.008
Filgotinib	15	3	0.6	0.12	0.024
Flavopiridol	2	0.4	0.08	0.016	0.0032
Fludarabine	10	2	0.4	0.08	0.016
Ganetespib	2	0.4	0.08	0.016	0.0032
Gilteritinib	15	3	0.6	0.12	0.024
I-BET-762	5	1	0.2	0.04	0.008
Ibrutinib	1	0.2	0.04	0.008	0.0016
Idarubicin	15	3	0.6	0.12	0.024
Idasanutlin (RS7388)	20	4	0.8	0.16	0.032
Idelalisib	2	0.4	0.08	0.016	0.0032
Imatinib	10	2	0.4	0.08	0.016
Iniparib	15	3	0.6	0.12	0.024
IRAK 4 Compound 26	5	1	0.2	0.04	0.008
IRAK-1/4 INHIBITOR I	10	2	0.4	0.08	0.016
Ixazomib	5	1	0.2	0.04	0.008
JAK3 Inhibitor I	15	3	0.6	0.12	0.024
Lenalidomide	5	1	0.2	0.04	0.008
LY3039478	10	2	0.4	0.08	0.016
MI-773	5	1	0.2	0.04	0.008
Midostaurin hydrate	15	3	0.6	0.12	0.024
Mitoxantrone	5	1	0.2	0.04	0.008

MLN-120B	15	3	0.6	0.12	0.024
Navitoclax	2	0.4	0.08	0.016	0.0032
Nelarabine	15	3	0.6	0.12	0.024
Nilotinib	10	2	0.4	0.08	0.016
NSC 652287	15	3	0.6	0.12	0.024
Nutlin-3a	20	4	0.8	0.16	0.032
Obatoclax mesylate	15	3	0.6	0.12	0.024
Olaparib	15	3	0.6	0.12	0.024
Onalespib	2	0.4	0.08	0.016	0.0032
ONO-4059	1	0.2	0.04	0.008	0.0016
OTX015	10	2	0.4	0.08	0.016
Pacritinib	15	3	0.6	0.12	0.024
Palbociclib	15	3	0.6	0.12	0.024
Panobinostat	0.4	0.08	0.016	0.0032	0.00064
Pentostatin	18.76	3.75	0.75	0.15	0.03
Pomalidomide	2	0.4	0.08	0.016	0.0032
Pralatrexate	15	3	0.6	0.12	0.024
Quizartinib	10	2	0.4	0.08	0.016
Resveratrol	15	3	0.6	0.12	0.024
Ribociclib	15	3	0.6	0.12	0.024
Rigosertib	5	1	0.2	0.04	0.008
Romidepsin	0.2	0.04	0.008	0.0016	0.00032
Roscovitine	20	4	0.8	0.16	0.032

Ruxolitinib	15	3	0.6	0.12	0.024
SCH772984	5	1	0.2	0.04	0.008
Selinexor	5	1	0.2	0.04	0.008
SGC 0946	15	3	0.6	0.12	0.024
Sorafenib	15	3	0.6	0.12	0.024
Temsirolimus	20	4	0.8	0.16	0.032
Thalidomide	20	4	0.8	0.16	0.032
Thioguanin	15	3	0.6	0.12	0.024
TMP269	15	3	0.6	0.12	0.024
Tofacitinib	15	3	0.6	0.12	0.024
Tozasertib	15	3	0.6	0.12	0.024
Trametinib	0.5	0.1	0.02	0.004	0.0008
UMI-77	15	3	0.6	0.12	0.024
Vemurafenib	15	3	0.6	0.12	0.024
Venetoclax	0.4	0.08	0.016	0.0032	0.00064
Vincristine	10	2	0.4	0.08	0.016
Vindesine	10	2	0.4	0.08	0.016
Vismodegib	15	3	0.6	0.12	0.024
Vorinostat	10	2	0.4	0.08	0.016

Supplementary Table S7: Drugs used in the validation drug screen.

Drug	Simplified drug class/ targeted pathway	Main targets/ mode of action	Supplier	Concentration 1 [µM]	Concentration 2 [µM]	Concentration 3 [µM]	Concentration 4 [µM]	Concentration 5 [µM]
Acacytidine	Chemotherapy	DNA Methylation	Biomol	20,00	4	0,8	0,16	0,032
BRD73954	Histone deacetylase	HDAC6, HDAC8	Selleck Chemicals	15,00	3	0,6	0,12	0,024
Cladribine	Chemotherapy	Purine analogue	Selleck Chemicals	15,00	3	0,6	0,12	0,024
Crenolanib	FLT3	FLT3	Selleck Chemicals	10,00	2	0,4	0,08	0,016
Crizotinib	HGF	MET, ALK	Selleck Chemicals	15,00	3	0,6	0,12	0,024
Cytarabin	Chemotherapy	DNA	Sigma-Aldrich	20,00	4	0,8	0,16	0,032
Daunorubicin	Chemotherapy	DNA, TOP2A, TOP2B	Selleck Chemicals	1,00	0,2	0,04	0,008	0,0016
Dinaciclib	CDK	CDK2, CDK5, CDK1, CDK9	Selleck Chemicals	0,04	0,008	0,0016	0,00032	0,000064
Fludarabine	Chemotherapy	POLA1, DNA	Selleck Chemicals	10,00	2	0,4	0,08	0,016
Gilteritinib	FLT3	FLT3	Selleck Chemicals	15,00	3	0,6	0,12	0,024
Ibrutinib	BCR	BTK	Selleck Chemicals	1,00	0,2	0,04	0,008	0,0016
Idarubicine	Chemotherapy	Topoisomerase II	Selleck Chemicals	3,00	0,6	0,12	0,024	0,0048
Midostaurin	PKC	PRKCA, PRKCG, PRKCB, KDR, SYK	Sigma-Aldrich	15,00	3	0,6	0,12	0,024

Mitoxant-rone	Chemotherapy	TOP2A, DNA	Sigma-Ald-rich	5,00	1	0,2	0,04	0,008
Navitoclax	Apoptosis	BCL2, BCL2L1, BCL2L2	Selleck Che-micals	2,00	0,4	0,08	0,016	0,0032
NSC 652287	DDR	MDM2, DNA	Selleck Che-micals	15,00	3	0,6	0,12	0,024
Quizartinib	FLT3	FLT3	Selleck Che-micals	10,00	2	0,4	0,08	0,016
SGC 0946	Histone methyl-transferase	DOT1L	Selleck Che-micals	15,00	3	0,6	0,12	0,024
Sorafenib	MAPK	RAF1, BRAF, KDR	Selleck Che-micals	15,00	3	0,6	0,12	0,024
UMI-77	Apoptosis	MCL1	Selleck Che-micals	15,00	3	0,6	0,12	0,024
Venetoclax	Apoptosis	BCL2	Selleck Che-micals	0,40	0,08	0,016	0,0032	0,00064
Vincristine	Chemotherapy	Vinca alkaloid	Selleck Che-micals	10,00	2	0,4	0,08	0,016
Vindesine	Chemotherapy	Vinca alkaloid	Sigma-Ald-rich	10,00	2	0,4	0,08	0,016

CLINICAL TRIAL PROTOCOL

Clinical trial code:

S*ystematic and* ***M****echanism-based* ***A****pproach to* ***R****ational* ***T****reatment Trial of Blood Cancer (SMARTrial)*

Study type: Observational study on biomarkers in **hematological cancers.**

CONFIDENTIAL

This protocol is confidential information and is intended solely for the guidance of the clinical investigation. This protocol may not be disclosed to parties not associated with the clinical investigation or used for any purpose without the prior written consent of the LKP.

Lead Investigator (LKP)<br>Sascha Dietrich, MD<br>Medizinische Klinik V<br>Im Neuenheimer Feld 410<br>69120 Heidelberg<br>Germany	Principal investigators<br>T. Zenz, MD, DKFZ<br>Axel Benner, DKFZ<br>Nora Liebers, MD, DKFZ<br>Wolfgang Huber, PhD, EMBL
Scientific lead<br>Thorsten Zenz, MD, DKFZ<br>Wolfgang Huber, PhD, EMBL	Data Management<br>Axel Benner, DKFZ<br>Wolfgang Huber, PhD, EMBL
Biometrician<br>Axel Benner, DKFZ	**Clinical Monitoring**<br>N.N.

Table of Contents

Table of Contents 3

Protocol Outline 5

Flow Chart 8

Abbreviations 9

1. Background and rationale 11
   1.1 Scientific Background 11
   1.2 Trial rational 11
   1.3 Benefit-Risk Assessment 12
2. Trial Objectives and Endpoints 13
   2.1 Objectives 13
   2.2 Endpoints 13
3. Clinical response definition 14
4. Trial design 15
5. Trial Duration and Schedule 15
6. Selection of Subjects 15
   6.1 Number of Subjects 15
   6.2 Inclusion and Exclusion Criteria 16
   6.3 Criteria for Withdrawal 17
   6.4 Replacement of Subjects 17
   6.5 Premature Closure of the Clinical Trial 17
7. Medical Device 18
   7.1 General Information about the Device 18
   7.2 Description of study test 18
8. Trial description and observation plan 19
   8.1 Description of Trial Schedule 19
   8.2 Methods of Data Collection 26
9. Ethical and Regulatory Requirements/ Quality Assurance 27
   9.1 Legal Framework 27
   9.2 Ethical Requirements 27
   9.3 Informed Consent 27
   9.4 Patient Privacy and Data Protection 28
10. Statistical Procedures 29

10.1 Sample Size Calculation 29
10.2 Analysis Variables 29
10.3 Statistical Methods 29
10.4 Interim Analyses 30
11. Data management 31
11.1 Data Collection 31
11.2 Data Handling 31
11.3 Storage and Archiving of Data 31
12. Ethical and Legal Aspects 32
12.1 Good Clinical Practice 32
12.2 Subject Information and Informed Consent 32
12.3 Confidentiality 32
12.4 Responsibilities of Investigator 32
12.5 Approval of Trial Protocol and Amendments 33
12.6 Continuous Information to Independent Ethics Committee 33
12.7 Advisory Board 33
13. Agreements 34
13.1 Financing of the Trial 34
13.2 Publication 34
14. Signatures 35
15. Declaration of Investigator 36
16. References 37

Protocol Outline

Aim:

The aim of this clinical research project is to evaluate if drug response testing can be performed within 7 days and to evaluate the value of *ex vivo* drug screening for hematological blood cancers as a biomarker to predict outcome, clinical course and response to treatment.

Study type: Observational study on biomarkers in hematological cancers.

Objectives:

a) Demonstrate feasibility of drug sensitivity testing of primary patient derived cancer cells.

b) Association of patients' response and ex-vivo response to drugs.

Primary endpoint:

a) Rate of successfully completed assessments of drug response to drugs/inhibitors within 7 days (non-interventional)

Key secondary endpoints:

a) Accuracy of patients' response prediction by *ex-vivo* drug response testing.

b) Prediction of time to next treatment within one year by *ex-vivo* drug response testing.

Trial design

Prospective, non-interventional (NIS), single-group, non-randomized, multi-center study.

Duration of trial phases: Recruitment of 80 patients within 24 months, drug-screened within 7 days after clinical visit and clinical course documented and follow-up of at least 12 months.

Population
Consecutive subjects with blood cancer in need of treatment, fulfilling all inclusion criteria will be considered eligible for enrolment.

Key inclusion criteria:

1) Diagnosis of a hematological malignancy: patients with leukemia, myeloma or lymphoma (e.g. ALL, AML, CLL, T-PLL, MCL, MM) who are in need of treatment and are willing to donate sufficient tumor material for ex-vivo drug sensitivity testing.
2) The treating physician needs to indicate treatment.
3) Measurable disease burden according to criteria as mention in **section 3**.
4) Treatment must be scheduled and the patient must be eligible for the planed treatment as judged by the treating physician.
5) Availability of $5x10^7$ cells from peripheral blood draws, bone marrow aspirations or lymph node biopsies.
6) Patient's written informed consent present.
7) Ability to understand the nature of the trial and the trial related procedures and to comply with them.

Key exclusion criteria:

1) Any condition, which precludes initiation of treatment (e.g. breast feeding, pregnancy, infections, etc.) as judged by the treating physician.
2) Any coexisting medical or psychological condition that would preclude participation in the required study procedures, as judged by the treating physician.
3) No systemic cancer treatment except for cytoreductive pretreatment within 1 week of enrollment

Trial methods

We measure pathway sensitivity (and resistance) of primary tumor cells *ex vivo* using a diverse compound library across blood cancers of patients in need of treatment. The drug library covers a spectrum of pathway inhibitors and state of the art drugs that can be prescribed or are currently in clinical trials. Fresh tumor material drawn from blood or bone marrow will be cultured and exposed to the panel of drugs *ex vivo*. Viability of primary tumor cells is assessed to determine sensitivity. Bioinformatic tools are used and developed to calculate sensitivity scores for each drug taking into account a reference sample set.

Patients' response to treatment will be assessed and correlated with *ex vivo* drug response data to understand correlation and the predictive power of the assay. Responses after individual treatments will be assessed according to standard procedures including response rate, clearance of tumor cells from blood and bone marrow.

Patients will be followed for 12 months after study entry.

Trial duration

Phase I: Recruitment of 80 patients within 24 months, screened within 7 days after clinical visit and clinical course documented and follow-up of at least 12 months.

Flow Chart

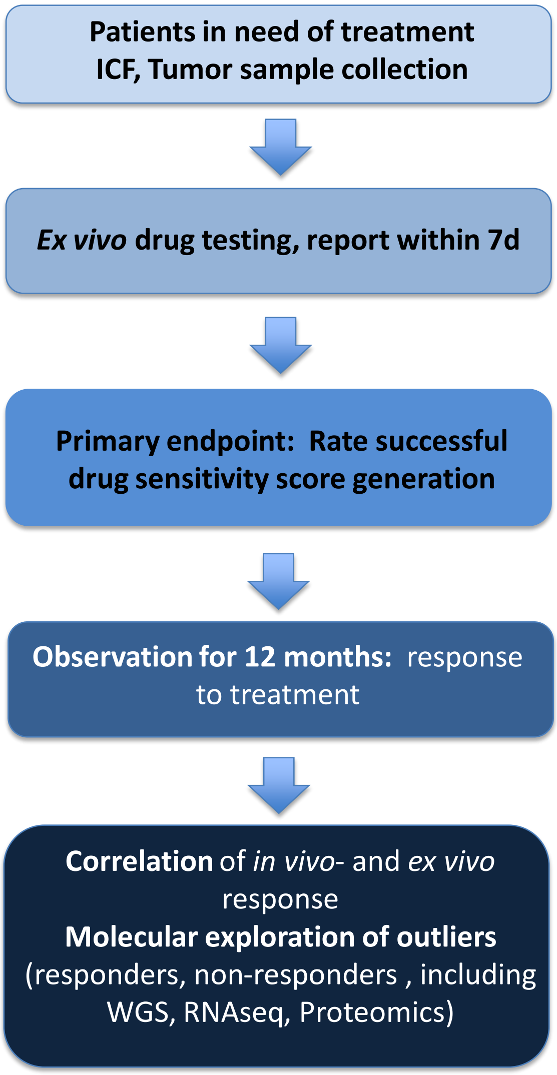

Patients in need of treatment
ICF, Tumor sample collection
Ex vivo drug testing, report within 7d
Primary endpoint: Rate successful drug sensitivity score generation
Observation for 12 months: response to treatment
Correlation of *in vivo*- and *ex vivo* response
Molecular exploration of outliers
(responders, non-responders , including WGS, RNAseq, Proteomics)

Abbreviations

List of Abbreviations

AE	Adverse Event
ALL	Acute lymphoblastic leukemia
AML	Acute myeloid leukemia
AUC	Area Under the Curve
BP	Blood Pressure
CLL	Chronic lymphocytic lymphoma
CR	Complete Remission
CRA	Clinical Research Associate (Monitor)
CT	Computed tomography
CTCAE	Common Terminology Criteria for Adverse Events
CTU	Clinical Trials Unit
CyA	Cyclosporin A
d	day
DFS	Disease Free Survival
DMSC	Data Monitoring and Safety Committee
DNA	Desoxyribonucleic acid
DRKS	Deutsches Register Klinischer Studien (German Clinical Trials Register)
ECOG	Eastern Cooperative Oncology Group
eCRF	electronic Case Report Form
EMA	European Medicines Agency
EudraCT	European Union Drug Regulating Authorities Clinical Trials
FL	Follicular lymphoma
FPFV	First Patient First Visit
FU	Follow Up
GCP-V	German Decree of 09-Aug-2004 on the Use of Good Clinical Practices
Hb	Hemoglobin
HR	Hazard Ratio
i.v.	Intravenous(ly)
ICF	Informed Consent Form
IEC	Independent Ethics Committee
Kg	Kilogram
LDH	Lactate Dehydrogenase
LKP	Leiter der klinischen Prüfung (Coordinating investigator, according to German Drug Law, § 4 [25] and § 40 AMG)
LPLV	Last Patient Last Visit
MCL	Mantle cell lymphoma
MedDRA	Medical Dictionary for Regulatory Activities
MMF	Mycophenolate mofetil
MPA	Mycophenolic Acid

MPG	Medicinal Device Law (Medizinproduktegesetz)
NCI	National Cancer Institute
NHL	Non-Hodgkin lymphoma
NRM	Non-Relapse Mortality
NYHA	New York Heart Association
OS	Overall Survival
p.o.	per os
PBSCT	Peripheral Blood Stem Cell Transplantation
PD	Progressive Disease
PHI	Protected Health Information
PLT	Platelets
PTCL	Peripheral T cell lymphoma
SAE	Serious Adverse Event
SAP	Statistical Analysis Plan
SDV	Source Data Verification
SOP	Standard Operating Procedure
SSC	Scientific Steering Committee
SUSAR	Suspected Unexpected Serious Adverse Reaction
TCL	T-cell lymphoma
T-PLL	T-prolymphocytic leukemia
TMF	Trial Master File
WHO	World Health Organization

1. Background and rationale

1.1 Scientific Background

Response to anti-cancer agents is heterogeneous and often restricted to groups of patients which are sometimes ill-defined, as mechanisms underlying response heterogeneity and biomarkers predicting response are not known[1,2]. Determinants of drug response have been mapped in immortalized cancer cell lines (http://www.cancerRxgene.org[3]; http://www.broadinstitute.org/ccle[4]; http://www.broadinstitute.org/ctrp [5], and recent technology improvements increased throughput [6] and used "near-complete" genetic profiles[7]. However, an ideal platform to integrate complex clonal compositions of individual patients underlying variable drug response would directly interrogate primary cancer cells. A unique feature of such direct use of patient cells is the potential to derive individualized therapeutic options for the donating patients[8-10] and the ability to pursue sensitivity signals, a strategy which has yielded novel genetic markers and drug repurposing opportunities based on individual patient observations[11-13].
Recent work suggested that drug response patterns derived from a panel of kinase inhibitors applied to a focused cohort of primary leukemias could predict pathway dependence while simultaneously identifying potential therapeutic options directly for the donating patients[8,9] In addition, the combination of genetic profiling and drug sensitivity analysis provided unique insight and potential impact on patient care[11] While extremely promising, substantial research is needed to increase the robustness of such findings and support them with sound biology-based understanding and clinical trial structures.

1.2 Trial rational

Targeted treatments have revolutionized care of individual diseases [14-17]. While a new generation of targeted drugs is emerging in leukemia and lymphoma[18-21] it remains clinical reality that most genetic information is not used for therapeutic stratification[22,23]. This is in part based on the shortcomings of traditional biomarker discovery within clinical trials, where throughput is limited in both, drug number and sample size. If it were possible to map the variable pathway dependencies and drug sensitivity patterns in individual patients it is likely to become an asset to identify genotype-phenotype associations, understand the underlying complexities of molecular networks and further precision medicine stratification.

To link clinical outcome and ex-vivo drug response assays, we systematically measure pathway sensitivity and resistance of primary tumor cells *ex-vivo* using a

diverse compound library for individual patients in need of treatment. By systematically analyzing *ex-vivo* drug response patterns, we group tumors functionally, by response phenotype. While for the purpose of this study selection of a specific treatment will not be based on ex-vivo drug response assays, we will prospectively collect clinical response- and follow-up data of patients in parallel.

The aim of the trial is to demonstrate the feasibility of *ex-vivo* sensitivity testing and relate ex vivo results to treatment outcome. The study is designed to proof this concept by 1) assessing drug sensitivity phenotypes in high through-put and real-time, 2) and following patient outcome. Association of drug sensitivity assays and clinical response data will provide first insights of how well systematic ex-vivo drug response assays can predict treatment outcome.

1.3 Benefit-Risk Assessment

Patients agree to donate 30-40 ml of peripheral blood or bone-marrow aspirate to conduct ex-vivo drug response assays. Bone marrow aspirations will only be performed if indicated for routine medical reasons. If a bone marrow aspiration is indicated, patients agree to donate additional 30 ml of bone marrow. Neither blood donation nor the additional donation of bone marrow is associated with a significant harm for the patient.

In addition, patients agree that their clinical history and follow-up data will be registered in a pseudonomized clinical data base. Upon patient’s request registered data can will be deleted from the database.

2. Trial Objectives and Endpoints

2.1 <u>Objectives</u>

a) Demonstrate feasibility of drug sensitivity testing of primary patient derived cancer cells.

b) Association of patients' response (Clinical response definition, Section 3) to drug treatment as defined by disease specific standard criteria and ex-vivo response to drugs.

2.2 <u>Endpoints</u>

<u>Primary endpoint:</u>

a) Rate of successfully completed assessments of drug response to drugs/inhibitors within 7 days (non-interventional)

<u>Key secondary endpoints:</u>

a) Accuracy of patients' drug response prediction by ex-vivo drug profiling

b) Prediction of time to next treatment within one year by ex-vivo drug response profiling. Time to next treatment is defined as the time from start of treatment at study inclusion until potential subsequent treatment.

3. Clinical response definition

At inclusion a main disease specific response parameter (see below 1 to 5) is defined:

A clinical response is defined as in **a.** and a stable disease as in **b.**

1. Blood counts:

    a. Normalization of either hemoglobin > 10 g/dl, platelets > 100/nl or neutrophils > 1/nl if these values were below these thresholds at start of therapy.

    b. No evidence of deterioration of blood counts below above mentioned thresholds for 12 weeks or further deterioration by at least 20% if blood counts were below above mentioned thresholds.

2. Immunophenotyping of malignant cells in the peripheral blood or bone marrow:

    a. Reduction of the malignant cell clone by at least 50% (as defined by flow cytometry).

    b. No evidence of progression for 12 weeks (increase of the malignant cell count by at least 50%).

3. Bone marrow aspiration, peripheral blood smear (cytology)/ trephine biopsy:

    a. Reduction of malignant cell clone by at least 50% (as defined by cytology, or immunohistochemistry)

    b. No evidence of progression for 8 weeks (no increase of the malignant cell count by at least 50%)

4. Clinically established biomarker (e.g. sCD25 in case of HCL or M Protein in case of MM/ LPL):

    a. Reduction of the pre-treatment biomarker levels by at least 50%.

    b. No evidence of biomarker increase by at least 30% compared to pre-treatment levels.

5. Organ/ tumor manifestation:

   a. Reduction of the tumor size by at least 30% (as defined by any imaging modality, e.g. CT scan or sonography)

   b. No evidence of progression for 8 weeks (no increase of the tumor size by at least 20%)

4. Trial design

Prospective, non-interventional (NIS), single-group, non-randomized, multi-center study.

5. Trial Duration and Schedule

Recruitment of 80 patients is expected to be completed within 2 years.
Recruitment of subjects will start in Q2/2018.

a) Inclusion of first subject (First subject in; FSI): Q2/2018

b) Inclusion of last subject (Last subject in; LSI): Q2/2020

c) Database Closure: Q3/2021

d) Statistical Analysis: Q3/4/2022

e) Report: Q1/2023

6. Selection of Subjects

Consecutive subjects with blood cancer in need of treatment, fulfilling all inclusion criteria will be considered eligible for enrolment.

6.1 Number of Subjects

Recruitment of 80 patients within 24 months, screened within 7 days after clinical visit and clinical course documented and follow-up of at least 12 months.

6.2 Inclusion and Exclusion Criteria

Key inclusion criteria:

a) Diagnosis of a hematological malignancy: patients with leukemia, myeloma or lymphoma (e.g. ALL, AML, CLL, T-PLL, MCL, MM) who are in need of treatment and are willing to donate sufficient tumor material for ex-vivo drug sensitivity testing.
b) The treating physician needs to indicate treatment.
c) Measurable disease burden according to criteria as mention in **section 3**.
d) Treatment must be scheduled and the patient must be eligible for the planed treatment as judged by the treating physician.
e) Availability of $5x10^7$ cells from peripheral blood draws, bone marrow aspirations or lymph node biopsies.
f) Patient's written informed consent present.
g) Ability to understand the nature of the trial and the trial related procedures and to comply with them.

Key exclusion criteria:

a) Any condition, which precludes initiation of treatment (e.g. breast feeding, pregnancy, infections, etc.) as judged by the treating physician.
b) Any coexisting medical or psychological condition that would preclude participation in the required study procedures, as judged by the treating physician.
c) No systemic cancer treatment except for cytoreductive pretreatment within 1 week of enrollment.

6.3 Criteria for Withdrawal

Subject may be withdrawn from the trial for the following reasons:

a) At their own request
b) If, in the investigator's opinion, continuation of the trial would be detrimental to the subject's well-being
c) If protocol violations occur that would not allow to evaluate the primary and secondary endpoints (e.g. inability to receive treatment as judged by the treating physician, lack of sufficient tumor cells to conduct ex-vivo drug response testing).
d) If the period of observation does not allow to determine response
e) The Principal Investigator decides about withdrawal of subjects from the clinical trial in case of occurrence of criteria mentioned above.

In all cases, the reason for withdrawal must be recorded in the CRF and in the subject's medical records. In case of withdrawal of a subject at his/her own request, as far as possible the reason should be asked for as extensively as possible and documented. The subject will be followed up as far as possible; all examinations scheduled for the final trial day will be intended to be performed on all subjects and documented.

6.4 Replacement of Subjects

If *ex-vivo* drug response assays were performed, but the subject's observation period was not sufficient to determine response to given treatment we intend to replace such patients.

6.5 Premature Closure of the Clinical Trial

The trial can be prematurely closed by the LKP in case new facts of finding on *ex vivo* drug screening and correlation with *in vivo* responses become available, which do not justify study continuation. The Ethics Committee (EC) must then be informed.

7. Medical Device

7.1 General Information about the Device

- Product Code: EnVision® Xcite Multilabel Reader
- Product Identification: not applicable
- Application Form: not applicable
- Manufacturer: PerkinElmer (Waltham, MA, USA)

7.2 Description of study test

Cell viability after drug exposure will be measured *ex vivo* using the CellTiter Glo assay (Promega, Fitchburg, WI, USA) according to the manufacturer's instructions.

In brief, 50µl of primary leukemia cells in 384-well plates will be incubated with up to 430 different compounds. After 48h, CellTiter Glo will be added and absorbance is measured on a PerkinElmer Envision plate reader. Viability will be calculated as % of solvent (DMSO) control.

8. Trial description and observation plan

8.1 Description of Trial Schedule

Patient’s blood cancer samples will be collected at study inclusion before treatment is initiated. Baseline and disease relevant information is collected at study entry (see below: before treatment).

During treatment follow-up, visits are scheduled by the treating physician. Planed and fixed visits are scheduled at:

1) Study inclusion.

2) Each week after study inclusion for 4 weeks.

3) At 3, 6 and 12 months.

4) After treatment was finished or changed.

No additional follow-up visits are necessary based on the study protocol. At follow up visits during treatment baseline laboratory values and clinical data is collected (see below: during treatment).

After treatment disease specific response criteria and responses are documented (see below: after treatment).

1) Before treatment at study inclusion

	**Baseline**
1	Date of trial enrollment, Patient ID
2	Age
3	Sex
4	Inclusion and exclusion criteria
**5**	**Diagnosis**
5.1	Hematological diagnosis
5.2	Date of initial diagnosis

**6.**	**Blood counts**
6.1	Leukocytes
6.2	Neutrophils
6.3	Hemoglobin
6.4	Platelets
6.5	Lymphocytes
6.6	Blasts
6.7	LDH
**7**	**FACS**
7.1	PB (malignant cell count)
7.2	KM (malignant cell count)
**8**	**Bone marrow aspiration/ PB smear (only in case of AML/ ALL)**
8.1	% of blasts
**8.2**	**Triphine biopsy**
8.3	Immune-phenotype
8.4	Population
8.5	% infiltration
**9**	**Biomarker**
9.1	Level of biomarker
9.2	Unit of biomarker
**10**	**Organ manifestation**

10.1	Organ manifestation
10.2	Examination
10.3	Manifestation
11	**Molecular parameters**
11.1	FISH
11.2	Karyotype
11.3	Tp53
11.4	Molecular genetics
12	**Pretreatment**
12.1	Pretreated
12.2	Lines of pretreatment
12.3	Treatment
12.4	Date of last treatment

2) During treatment (each week after study inclusion for 4 weeks and at 3, 6 and 12 months).

	**Treatment**
1	Treatment
2	Specify treatment
3	Date start treatment
4	Treatment ongoing
5	Response

**6.**	**Blood counts**
6.1	Leukocytes
6.2	Neutrophils
6.3	Hemoglobin
6.4	Platelets
6.5	Lymphocytes
6.6	Blasts
6.7	LDH
**7**	**FACS**
7.1	PB (malignant cell count)
7.2	KM (malignant cell count)
**8**	**Bone marrow aspiration/ PB smear (only in case of AML/ ALL)**
8.1	% of blasts
**8.2**	**Triphine biopsy**
8.3	Immune-phenotype
8.4	Population
8.5	% infiltration
**9**	**Biomarker**
9.1	Level of biomarker
9.2	Unit of biomarker

**10**	**Organ manifestation**
10.1	Organ manifestation
10.2	Examination
10.3	Manifestation

3) End or change of treatment

1	Date of assessment
2	End of treatment date
3	Reason for treatment ending
4	Therapy duration
5	Number of administered cycles
6	New treatment
7	Response
**8.**	**Blood counts**
8.1	Leukocytes
8.2	Neutrophils
8.3	Hemoglobin
8.4	Platelets
8.5	Lymphocytes
8.6	Blasts
8.7	LDH

**9**	**FACS**
9.1	PB (malignant cell count)
9.2	KM (malignant cell count)
**10**	**Bone marrow aspiration/ PB smear (only in case of AML/ ALL)**
10.1	% of blasts
**10.2**	**Triphine biopsy**
10.3	Immune-phenotype
10.4	Population
10.5	% infiltration
**11**	**Biomarker**
11.1	Level of biomarker
11.2	Unit of biomarker
**12**	**Organ manifestation**
12.1	Organ manifestation
12.2	Examination
12.3	Manifestation

4) In case of death

1	Date of death
2	Cause of death

3	Classification of cause of death
4	Specification in case of other malignance
5	Relationship of death to last treatment

8.2 Methods of Data Collection

8.2.1 Blood processing:

Blood will be processed at the day of sampling. PBMC from full blood in EDTA will be separated using a Ficoll density gradient and frozen in freezing medium (900 µl of fetal calf serum and 100 µl of DMSO). The samples will be collected in the two centers and then send to laboratory of PD Dr. Sascha Dietrich.

Address:

Caroline Kolb

OMZ, Raum 1.004

Im Neuenheimer Feld 350

69120 Heidelberg

Germany

8.2.2 Case report forms

An electronic CRF form will be used to collect the data. The eCRF form was developed with the Onkostar software.

8.2.3 Reporting of Adverse Events

As this is a non-interventional, observational trial no adverse events related to this study will occur.

9. Ethical and Regulatory Requirements/ Quality Assurance

9.1 Legal Framework

This registry is not a clinical study according to the German Drug Law.

9.2 Ethical Requirements

This observational plan and the planned conduct of the registry have been submitted to the Ethic Committee (EC) of the University of Heidelberg. The EC Heidelberg approved the project and confirmed that it is not necessary to get the approval according to AMG 40-42 and the GCP-guidelines.

The registry project will be presented to the local ethic committees of the participating sites. The protocol for this registry has been designed in accordance with the general ethical principles outlined in the Declaration of Helsinki.

The approval for both the protocol and informed consent must specify the date of approval, protocol number and version, or amendment number.

9.3 Informed Consent

The investigator assumes the responsibility of obtaining written informed consent for each patient or the patient's legally authorized representative before enrolment of the patient in the registry. The Sponsor and the investigators affirm and uphold the principle of the patient's right to privacy. The investigators shall comply with applicable privacy laws. The investigator must assure that the patient's anonymity will be maintained and that the identities are protected from unauthorized parties. Patients meeting the criteria set forth in the protocol will be offered the opportunity to participate in the registry. To avoid introduction of bias, the investigator must exercise no selectivity with regard to offering eligible patients the opportunity to participate in the study. Patients will be given the opportunity to ask questions concerning the study, and adequate time to consider their decision to or not to participate. Informed consent will be documented by the use of a written consent form that includes all the elements required by regulations and ICH guidelines. The form has to be signed and dated by the patient or patient's legally authorized representative and by the person who administers the consent process. A copy of the signed form will be given to the person who signed it; another copy needs to be filed with the patient's medical records. The original signed consent form will be filed in the Investigator's site file. The date and time of the informed consent must additionally be recorded in the source documents.

9.4 Patient Privacy and Data Protection

The investigator affirms and upholds the principle of the patient’s right to privacy. The investigator shall comply with applicable privacy laws. The investigator must assure that the patient’s anonymity will be maintained and that the identities are protected from unauthorized parties. On CRFs and other documents patients should not be identified by their names. All clinical and scientific data as well as samples are collected under a code (or “pseudonym”), and stored in the main clinical trial database or bio-bank, respectively. Collected samples will be assigned a unique numeric identifier (code) with password protected access for selected personnel in a non-public server-based database. Information about the study sites is stored in a second separate and secret data file linked to the center code. By keeping these two additional data files secret and separate from the main clinical trial database, it is ensured that one cannot trace the identity of the treated individual. All data exchange with the study center is made solely via the code. All participating study centers are obliged to keep a secret patient identification log.

10. Statistical Procedures

10.1 Sample Size Calculation

Sample size calculation is based on the approach described in Li & Fine for testing sensitivity[24]. A total sample size of n=80 patients achieves 80% power to detect a change in sensitivity from 0.5 to 0.8 using a one-sided test with significance level $\alpha=0.05$. Furthermore, a sample size of n=80 would allow to test the one-sided null hypothesis that the correlation between *ex vivo* drug response and *in vivo* treatment response is not larger than 0.5 using significance level $\alpha=0.05$ with more than 80% power when the sample correlation is at least 0.7. We consider a high sensitivity and an *ex vivo* / *in vivo* correlation larger than 0.5 to be a necessary condition for considering a subsequent clinical trial.

In total n=80 patients will be considered for the trial assuming that 20% of them will not be eligible.

10.2 Analysis Variables

The proposed study will be used to prepare a clinical trial, which will formally test the use of drug sensitivity testing to guide treatment decisions. Therefore we want to determine the rate of successful *ex vivo* drug testing and gain insight into potential rates of therapy recommendations which can be made based on *ex vivo* drug response data. Correlation of in vivo treatment response with *ex vivo* drug response will be used to develop case number calculations required for a subsequent clinical trial. While not a formal part of the trial, n=1 experiments are envisioned based on clinical needs[14,25].

The main outcomes (rate of successful therapy recommendations that can be made based on drug response, predictive accuracy of *ex vivo* screening to predict patient's treatment response) have not been tested in trials.

The *ex vivo* drug screen has been validated across centers (collaboration with FIMM, Helsinki) and across testing platforms and operators with high reproducibility. Validation of the feasibility of the approach is a major aim of the trial.

10.3 Statistical Methods

To evaluate diagnostic accuracy of *ex vivo* drug response with respect to treatment response sensitivity and specificity measures will be computed. Based on the correlation analysis of measures of *in vivo* treatment response and *ex vivo* drug

response a drug-sensitivity scoring system will be developed. The scoring system will be evaluated by computing the predictive accuracy of *in vivo* response to investigator chosen treatment using bootstrap re-sampling. In addition, PFS estimates will be computed considering drug-sensitivity scores.

If a successful scoring system could be derived we aim to perform a subsequent prospective clinical trial, using the platform to guide patient treatment.

10.4 Interim Analyses

Not planned.

11.Data management

11.1 Data Collection

All findings will be documented in the subject's medical record. All screening measures and the end-of-study measures are recorded on eCRF. All findings in the course of the study will be documented in customized eCRF (Oncostar). The investigator is responsible for ensuring that all sections of the eCRF are completed correctly and that entries can be verified against source data, where source data are available. Printed copies of the source data will be stored at the study office, and correctness of source data is verified by the treating- or study physician by his or her signature. Drug sensitivity data are stored as a csv file.

11.2 Data Handling

All data will be entered in a database as recorded in the eCRFs. All information in CRFs will be transferred automatically into CSV tables. After completion of data entry checks for plausibility, consistency and completeness of the data will be performed. Based on this checks, queries will be produced combined with the queries generated by visual control. All missing data or inconsistencies will be reported back to the center and clarified with a responsible investigator. If no further corrections are to be made in the database it will be declared closed and used for statistical analysis.

11.3 Storage and Archiving of Data

The investigator will archive all trial data (subject identification code list, source data and investigator's file) and relevant correspondence in the Investigator Site File (ISF). The ISF, all source data and all documents itemized in section 8 of the ICH Consolidated Guideline on GCP will be archived after finalization of the trial according to the legal regulations.

12.Ethical and Legal Aspects

12.1 Good Clinical Practice

The procedures set out in this trial protocol, pertaining to the conduct, evaluation, and documentation of this trial, are designed to ensure that all persons involved in the trial abide by Good Clinical Practice (GCP) and the ethical principles described in the current revision of the Declaration of Helsinki. The trial will be carried out in keeping with local legal and regulatory requirements.

12.2 Subject Information and Informed Consent

Before being admitted to the clinical trial, the subject will be asked to consent to participate after the nature, scope, and possible consequences of the clinical trial have been explained in a form understandable to him or her. The subjects will be asked to give consent in writing. Only those subjects who gave an informed consent may be included in the study. A copy of the signed informed consent document will be given to the subject. The documents must be in a language understandable to the subject and must specify who informed the subject.

12.3 Confidentiality

During the clinical trial, subjects will be identified solely by means of an individual identification code (subject number). Trial findings stored on a computer will be stored in accordance with local data protection law and will be handled in strictest confidence. For protection of these data, organizational procedures are implemented to prevent distribution of data to unauthorized persons. The appropriate regulations of local data legislation will be fulfilled in its entirety. Authorized persons (e.g. clinical monitors) may inspect the subject-related data collected during the trial ensuring the data protection law. The investigator will maintain a personal subject identification list (subject numbers with the corresponding subject names) to enable records to be identified.

12.4 Responsibilities of Investigator

The investigator should ensure that all persons assisting with the trial are adequately informed about the protocol, any amendments to the protocol, the trial treatments, and their trial-related duties and functions. The investigator should maintain a list of

subinvestigators and other appropriately qualified persons to whom he or she has delegated significant trial-related duties.

12.5 Approval of Trial Protocol and Amendments

Before the start of the trial, the trial protocol, informed consent document, and any other appropriate documents will be submitted to the independent registered Ethics Committee (EC). Formal approval by the EC should preferably mention the title of the trial, the trial code, the trial site, and any other documents reviewed. It must mention the date on which the decision was made and must be officially signed by a committee member. This documentation must also include a list of members of the EC present on the applicable EC meeting. Before the first subject is enrolled in the trial, all ethical and legal requirements will be met. Neither the investigator nor the KKS Heidelberg will alter this trial protocol without obtaining the written agreement of the other. The EC must be informed of all protocol amendments. The investigator must keep a record of all communications with the EC and the regulatory authorities.

12.6 Continuous Information to Independent Ethics Committee

The EC must be informed of all subsequent protocol amendments which require formally approval in accordance with local legal requirements.

The EC must be informed of trial process regularly if not otherwise stated in the vote.

The EC must be informed of the end of the trial.

12.7 Advisory Board

An Advisory Board will not be established.

13. Agreements

13.1 Financing of the Trial

This trial is sponsored by the National Center for Tumor Diseases.

13.2 Publication

All information concerning the trial is confidential before publication. The results of the trial will be published in a peer-reviewed journal.

14. Signatures

The present trial protocol was subject to critical review and has been approved in the present version by the persons undersigned. The information contained is consistent with:

- the current risk-benefit assessment.
- the moral, ethical, and scientific principles governing clinical research as set out in the Declaration of Helsinki and the principles of GCP.

The investigator will be supplied with details of any significant or new finding including adverse incidents.

Date __24.04.2018_ Signature: ______________________
Name (block letters): ______________________
Function: Sascha Dietrich
Clinical coordinating Investigator

Date __15.12.2015_ Signature: ______________________
Name (block letters): ______________________
Function: Axel Benner
Biometrician

Date __24.04.2018_ Signature: ______________________
Name (block letters): ______________________
Function: Nora Liebers
Trial coordinator Heidelberg

Date __15.12.2015_ Signature: ______________________
Name (block letters): ______________________
Function: Thorsten Zenz
Scientific coordinator

Date __15.12.2015_ Signature: ______________________
Name (block letters): ______________________
Function: Wolfgang Huber
Scientific coordinator

15.Declaration of Investigator

I have read the above trial protocol and I confirm that it contains all information to accordingly conduct the clinical trial. I pledge to conduct the clinical trial according to the protocol.

I will enroll the first subject only after all ethical requirements are fulfilled. I pledge to obtain written consent for trial participation from all subjects.

I know the requirements for accurate notification of adverse incidents and I pledge to document and notify such events as described in the protocol.

I pledge to retain all trial-related documents and source data as described. I will provide a Curriculum Vitae (CV) before trial start. I agree that the CV may be submitted to the responsible regulatory authorities.

Date ____________

Signature: ______________________________

Name (block letters): ______________________________

Function: Investigator

Trial center (address): ______________________________

____

____

16.References

1. de Gramont A, Watson S, Ellis LM, et al. Pragmatic issues in biomarker evaluation for targeted therapies in cancer. Nature Reviews Clinical Oncology 2015;12:197-212.
2. Sawyers CL. The cancer biomarker problem. Nature 2008;452:548-52.
3. Garnett MJ, Edelman EJ, Heidorn SJ, et al. Systematic identification of genomic markers of drug sensitivity in cancer cells. Nature 2012;483:570-5.
4. Barretina J, Caponigro G, Stransky N, et al. The Cancer Cell Line Encyclopedia enables predictive modelling of anticancer drug sensitivity. Nature 2012;483:603-7.
5. Basu A, Bodycombe NE, Cheah JH, et al. An interactive resource to identify cancer genetic and lineage dependencies targeted by small molecules. Cell 2013;154:1151-61.
6. Yu C, Mannan AM, Yvone GM, et al. High-throughput identification of genotype-specific cancer vulnerabilities in mixtures of barcoded tumor cell lines. Nat Biotechnol 2016;34:419-23.
7. Iorio F, Knijnenburg TA, Vis DJ, et al. A Landscape of Pharmacogenomic Interactions in Cancer. Cell 2016;166:740-54.
8. Tyner JW, Yang WF, Bankhead A, et al. Kinase pathway dependence in primary human leukemias determined by rapid inhibitor screening. Cancer research 2013;73:285-96.
9. Pemovska T, Kontro M, Yadav B, et al. Individualized systems medicine strategy to tailor treatments for patients with chemorefractory acute myeloid leukemia. Cancer discovery 2013;3:1416-29.
10. Crystal AS, Shaw AT, Sequist LV, et al. Patient-derived models of acquired resistance can identify effective drug combinations for cancer. Science 2014;346:1480-6.
11. Maxson JE, Gotlib J, Pollyea DA, et al. Oncogenic CSF3R Mutations in Chronic Neutrophilic Leukemia and Atypical CML. New England Journal of Medicine 2013;368:1781-90.
12. Pemovska T, Johnson E, Kontro M, et al. Axitinib effectively inhibits BCR-ABL1(T315I) with a distinct binding conformation. Nature 2015;519:102-5.
13. Maxson JE, Abel ML, Wang J, et al. Identification and Characterization of Tyrosine Kinase Nonreceptor 2 Mutations in Leukemia through Integration of Kinase Inhibitor Screening and Genomic Analysis. Cancer Research 2016;76:127-38.
14. Dietrich S, Glimm H, Andrulis M, von Kalle C, Ho AD, Zenz T. BRAF inhibition in refractory hairy-cell leukemia. N Engl J Med 2012;366:2038-40.
15. Druker BJ, Sawyers CL, Kantarjian H, et al. Activity of a specific inhibitor of the BCR-ABL tyrosine kinase in the blast crisis of chronic myeloid leukemia and acute lymphoblastic leukemia with the Philadelphia chromosome. N Engl J Med 2001;344:1038-42.
16. Flaherty KT, Puzanov I, Kim KB, et al. Inhibition of mutated, activated BRAF in metastatic melanoma. N Engl J Med 2010;363:809-19.
17. Huang ME, Ye YC, Chen SR, et al. Use of all-trans retinoic acid in the treatment of acute promyelocytic leukemia. Blood 1988;72:567-72.
18. Byrd JC, Furman RR, Coutre SE, et al. Targeting BTK with Ibrutinib in Relapsed Chronic Lymphocytic Leukemia. New England Journal of Medicine 2013;369:32-42.

19. Furman RR, Sharman JP, Coutre SE, et al. Idelalisib and rituximab in relapsed chronic lymphocytic leukemia. N Engl J Med 2014;370:997-1007.
20. Roberts AW, Davids MS, Pagel JM, et al. Targeting BCL2 with Venetoclax in Relapsed Chronic Lymphocytic Leukemia. New England Journal of Medicine 2015;374:311-22.
21. Wang ML, Rule S, Martin P, et al. Targeting BTK with Ibrutinib in Relapsed or Refractory Mantle-Cell Lymphoma. New England Journal of Medicine 2013;369:507-16.
22. Landau DA, Tausch E, Taylor-Weiner AN, et al. Mutations driving CLL and their evolution in progression and relapse. Nature 2015;526:525-30.
23. Puente XS, Beà S, Valdés-Mas R, et al. Non-coding recurrent mutations in chronic lymphocytic leukaemia. Nature 2015;526:519-24.
24. Li J, Fine J. On sample size for sensitivity and specificity in prospective diagnostic accuracy studies. Stat Med 2004;23:2537-50.
25. Dietrich S, Hullein J, Hundemer M, et al. Continued Response Off Treatment After BRAF Inhibition in Refractory Hairy Cell Leukemia. J Clin Oncol 2013;31:e300-3.
