# Supplementary material for: Ex vivo drug response profiling for response and outcome prediction in hematologic malignancies: the prospective non-interventional SMARTrial
Source: Nat Cancer. 2023 Oct 2;4(12):1648–59. doi: 10.1038/s43018-023-00645-5 (PMC10733146; doi:10.1038/s43018-023-00645-5)
Supplement: Supplementary file 2 — Reporting Summary [file 43018_2023_645_MOESM2_ESM.pdf]

nature portfolio

Corresponding author(s): Sascha Dietrich

Last updated by author(s): Aug 23, 2023

Reporting Summary

Nature Portfolio wishes to improve the reproducibility of the work that we publish. This form provides structure for consistency and transparency in reporting. For further information on Nature Portfolio policies, see our Editorial Policies and the Editorial Policy Checklist.

Statistics

For all statistical analyses, confirm that the following items are present in the figure legend, table legend, main text, or Methods section.

n/a	Confirmed	
☐	☒	The exact sample size (*n*) for each experimental group/condition, given as a discrete number and unit of measurement
☐	☒	A statement on whether measurements were taken from distinct samples or whether the same sample was measured repeatedly
☐	☒	The statistical test(s) used AND whether they are one- or two-sided<br>*Only common tests should be described solely by name; describe more complex techniques in the Methods section.*
☐	☒	A description of all covariates tested
☐	☒	A description of any assumptions or corrections, such as tests of normality and adjustment for multiple comparisons
☐	☒	A full description of the statistical parameters including central tendency (e.g. means) or other basic estimates (e.g. regression coefficient) AND variation (e.g. standard deviation) or associated estimates of uncertainty (e.g. confidence intervals)
☐	☒	For null hypothesis testing, the test statistic (e.g. *F*, *t*, *r*) with confidence intervals, effect sizes, degrees of freedom and *P* value noted<br>*Give P values as exact values whenever suitable.*
☒	☐	For Bayesian analysis, information on the choice of priors and Markov chain Monte Carlo settings
☒	☐	For hierarchical and complex designs, identification of the appropriate level for tests and full reporting of outcomes
☐	☒	Estimates of effect sizes (e.g. Cohen's *d*, Pearson's *r*), indicating how they were calculated

Our web collection on statistics for biologists contains articles on many of the points above.

Software and code

Policy information about availability of computer code

Data collection	Clinical data were recorded in an electronic case report form (eCRF) using Onkostar (IT-Choice Software AG, Version 2.8.0). Ex vivo drug response data was measured with an EnSight Multimode Plate Reader (Perkin Elmer).
Data analysis	All statistical analyses were performed in R version 4.1.3 (R Foundation for Statistical Computing, Vienna, Austria). The exact analysis packages used and the respective version information is listed in the the analysis markdown file (https://github.com/PeterBruch/SMARTrial/blob/main/inst/doc/SMARTrial_Analysis.html)

For manuscripts utilizing custom algorithms or software that are central to the research but not yet described in published literature, software must be made available to editors and reviewers. We strongly encourage code deposition in a community repository (e.g. GitHub). See the Nature Portfolio guidelines for submitting code & software for further information.

Data

Policy information about availability of data

All manuscripts must include a data availability statement. This statement should provide the following information, where applicable:

- Accession codes, unique identifiers, or web links for publicly available datasets
- A description of any restrictions on data availability
- For clinical datasets or third party data, please ensure that the statement adheres to our policy

In vivo and ex vivo response data as well as patient annotations are available at https://github.com/PeterBruch/SMARTrial. The data can also be interactively

explored through our web application at https://www.dietrichlab.de/SMARTrial/.
Source data for all main and Extended Data Figures is supplied with the submission. Whole-Exome-Sequencing data obtained for the AML validation cohort is available at the European Genome-Phenome Archive under accession number EGAS00001007223.

Human research participants

Policy information about studies involving human research participants and Sex and Gender in Research.

Reporting on sex and gender

Sex and Gender were not part of the inclusion or exclusion criteria. Self-reported sex was documented at study inclusion and is listed for all patients in Supplementary Table S1. Patient characteristics, including sex, are shown in Table 1.

Population characteristics

Patient characteristics are shown in Table 1 in an aggregated form and in Supplementary Table S1 in a disaggregated form.

SMARTrial patient cohort:

Sex : Male 48 (60 %), Female 32 (40 %)

Age (years): Median [Min, Max] 68.5 [20.0, 91.0]

Diagnosis:
AML 34 (42 %)
ALL 2 (2 %)
DLBCL 4 (5 %)
Burkitt lymphoma 1 (1 %)
Follicular lymphoma 3 (4 %)
Mantle cell lymphoma 5 (6 %)
CLL 25 (31 %)
B-PLL 1 (1 %)
T-PLL 4 (5 %)
T-cell lymphoma, NOS 1 (1 %)

AML Validation cohort:
Sex: Female 47 (49 %), Male 48 (51 %)
Age (years): Median [Min, Max] 59.0 [18.0, 84.0]

Recruitment

Adult patients with a diagnosis of a hematologic malignancy in need of treatment and willing to donate sufficient tumor material were eligible. Additional eligibility criteria included measurable disease burden for response assessment and the availability of at least 5x107 cells from peripheral blood draws, bone marrow aspirations or lymph node biopsies. Systemic cancer treatment other than cytoreductive pretreatment within 7 days before enrollment was an exclusion criterion. Complete inclusion and exclusion criteria are available in the study protocol (see Appendix).

For the validation cohort, we investigated the drug response of 95 AML samples obtained from the AML-biobank of the Study Alliance Leukemia (EK 98032010). All patients consented to biobanking and sample use for research projects.

Ethics oversight

The study was approved by the ethics committee of the University of Heidelberg, Heidelberg, Germany (S-683/2016) and conducted in accordance with the Declaration of Helsinki. All patients provided written informed consent.

Note that full information on the approval of the study protocol must also be provided in the manuscript.

Field-specific reporting

Please select the one below that is the best fit for your research. If you are not sure, read the appropriate sections before making your selection.

☒ Life sciences ☐ Behavioural & social sciences ☐ Ecological, evolutionary & environmental sciences

For a reference copy of the document with all sections, see nature.com/documents/nr-reporting-summary-flat.pdf

Life sciences study design

All studies must disclose on these points even when the disclosure is negative.

Sample size

Initial Sample size calculation as per study protocol:

Sample size calculation is based on the approach described in Li & Fine for testing sensitivity. A total sample size of n=80 patients achieves 80% power to detect a change in sensitivity from 0.5 to 0.8 using a one-sided test with significance level α=0.05. Furthermore, a sample size of n=80 would allow to test the one-sided null hypothesis that the correlation between ex vivo drug response and in vivo treatment response is not larger than 0.5 using significance level α=0.05 with more than 80% power when the sample correlation is at least 0.7. We consider a high

	sensitivity and an ex vivo / in vivo correlation larger than 0.5 to be a necessary condition for considering a subsequent clinical trial. In total n=80 patients will be considered for the trial assuming that 20% of them will not be eligible.
Data exclusions	All 80 patients included in the study were evaluated for the primary endpoint. For the secondary endpoint analyses 16 patients were excluded due to :<br>• Reduced quality of ex vivo screen (n= 2)<br>• In vivo response not evaluable (n=9)<br>• Other (n=5)<br>Details are shown in Figure 2.
Replication	Ex vivo screening data was reproducible using technical replicates of viably frozen tumor cells. For two patient samples, viably frozen cells were thawed after completion of the initial assay and investigated as technical replicates. These samples showed high reproducibility (R>0.9). These replicates were not used for the downstream data analysis.
Randomization	No randomization was performed. Covariates, most importantly disease entity, was considered for secondary endpoint analyses by performing subgroup analyses.
Blinding	Group allocation was based on disease entity and treatment assigned by the treating physician. Physicians were not blinded as treatment was based on physicians choice. Results of ex vivo drug response profiling used for treatment stratification only in an individual case of treatment failure to initial treatment.

Reporting for specific materials, systems and methods

We require information from authors about some types of materials, experimental systems and methods used in many studies. Here, indicate whether each material, system or method listed is relevant to your study. If you are not sure if a list item applies to your research, read the appropriate section before selecting a response.

Materials & experimental systems

n/a	Involved in the study
☒	☐ Antibodies
☒	☐ Eukaryotic cell lines
☒	☐ Palaeontology and archaeology
☒	☐ Animals and other organisms
☐	☒ Clinical data
☐	☒ Dual use research of concern

Methods

n/a	Involved in the study
☒	☐ ChIP-seq
☒	☐ Flow cytometry
☒	☐ MRI-based neuroimaging

Clinical data

Policy information about clinical studies
All manuscripts should comply with the ICMJE guidelines for publication of clinical research and a completed CONSORT checklist must be included with all submissions.

Clinical trial registration	NCT03488641
Study protocol	The study protocol is available in the Supplementary material.
Data collection	Demographic and clinical data were collected at study entry. During treatment and the post-treatment phase, patients were regularly followed up (at weeks 1-4 and at months 3, 6 and 12, after treatment cessation or change) for at least 1 year after study entry. Clinical data were recorded in an electronic case report form (eCRF) using Onkostar (IT-Choice Software AG).<br><br>Clinical data was obtained during treatment appointments at the University Hospital Heidelberg.
Outcomes	Clinical response characteristics have been predefined. See study protocol page 14&15.<br><br>Primary endpoint:<br>a) Rate of successfully completed assessments of drug response to drugs/inhibitors within 7 days (non-interventional)<br><br>Key secondary endpoints:<br>a) Accuracy of patients response prediction by ex-vivo drug response testing.<br>b) Prediction of time to next treatment within one year by ex-vivo drug response testing.

Dual use research of concern

Policy information about dual use research of concern

Hazards

Could the accidental, deliberate or reckless misuse of agents or technologies generated in the work, or the application of information presented in the manuscript, pose a threat to:

No	Yes	
☒	☐	Public health
☒	☐	National security
☒	☐	Crops and/or livestock
☒	☐	Ecosystems
☒	☐	Any other significant area

Experiments of concern

Does the work involve any of these experiments of concern:

No	Yes	
☒	☐	Demonstrate how to render a vaccine ineffective
☒	☐	Confer resistance to therapeutically useful antibiotics or antiviral agents
☒	☐	Enhance the virulence of a pathogen or render a nonpathogen virulent
☒	☐	Increase transmissibility of a pathogen
☒	☐	Alter the host range of a pathogen
☒	☐	Enable evasion of diagnostic/detection modalities
☒	☐	Enable the weaponization of a biological agent or toxin
☒	☐	Any other potentially harmful combination of experiments and agents
